# Ncl1-dependent m^5^C RNA methylation promotes appressorial autophagy and pathogenicity in the rice blast fungus

**DOI:** 10.1016/j.xplc.2026.101817

**Published:** 2026-03-11

**Authors:** Xuan Cai, Hong Hu, Caiyun Liu, Zhiyong Ren, Yunfeng Li, Yanfang Nie, Hao Liu, Lu Zheng, Junbin Huang, Xiaolin Yang, Xiao-Lin Chen

**Affiliations:** 1 Hubei Key Laboratory for Crop Diseases, Insect Pests, and Weeds Control, Key Laboratory of Integrated Pest Management on Crops in Central China of the Ministry of Agriculture and Rural Affairs, Institute of Plant Protection and Soil Science, Hubei Academy of Agricultural Sciences, Wuhan, China; 2 State Key Laboratory of Agricultural Microbiology, Provincial Key Laboratory of Plant Pathology of Hubei Province, College of Plant Science and Technology, Huazhong Agricultural University, Wuhan 430070, China; 3 Guangdong Provincial Key Laboratory of High Technology for Plant Protection, Plant Protection Research Institute, Guangdong Academy of Agricultural Sciences, Guangzhou 510640, China; 4 Guangdong Province Key Laboratory of Microbial Signals and Disease Control, College of Plant Protection, South China Agricultural University, Guangzhou 510642, China; 5 Hubei Hongshan Laboratory, Wuhan 430070, China

**Keywords:** 5-methylcytosine, RNA methylation, *Magnaporthe oryzae*, appressorium formation, autophagy

## Abstract

Epigenetic regulation, particularly RNA methylation, remains largely unexplored in plant-pathogenic fungi. This study investigates the role of 5-methylcytosine (m^5^C) RNA methylation during infection by *Magnaporthe oryzae*, the rice blast fungus, with an emphasis on its effects on autophagy. We identified Ncl1, an m^5^C RNA methyltransferase, as essential for fungal virulence and appressorium development. Using m^5^C RNA immunoprecipitation sequencing, we mapped the m^5^C landscape of the *M. oryzae* transcriptome, identifying 9014 hypomethylated peaks across 5678 genes in the Δ*ncl1* mutant. RNA sequencing analysis revealed that 119 m^5^C-modified genes were upregulated and 199 were downregulated, indicating a key role for Ncl1 in regulating gene expression. Mechanistically, Ncl1-mediated m^5^C methylation stabilizes *ATG5* and *ATG16* mRNAs, which are essential for Atg8 lipidation, autophagosome formation, and full *M. oryzae* pathogenicity. Notably, Ncl1 protein stability is modulated by the Pmk1 signaling pathway through phosphorylation and Smt3-mediated SUMOylation. These findings reveal a complex interplay between epigenetic regulation and post-translational modifications in fungi. They highlight the central role of m^5^C RNA methylation in autophagy and pathogenicity in *M. oryzae*, enhance our understanding of fungal biology, and provide potential targets for antifungal strategies.

## Teaser

This study reports that Ncl1-mediated m^5^C RNA methylation stabilizes ATG5 and ATG16 mRNAs in *Magnaporthe oryzae*, thereby promoting Atg8 lipidation and appressorial autophagy. Ncl1 protein stability is regulated by Pmk1-mediated phosphorylation and Smt3-dependent SUMOylation. This epigenetic regulatory axis is essential for fungal pathogenicity and provides a novel target for rice blast disease control.

## Introduction

Post-transcriptional RNA modifications have emerged as a cornerstone of epigenetic regulation, with over 100 chemically distinct modifications identified to date, including N6-methyladenosine (m^6^A), N1-methyladenosine (m^1^A), and 5-methylcytosine (m^5^C) (Machnicka et al., 2013). Although the roles of m^6^A in mRNA metabolism and cellular homeostasis are well documented (Roundtree et al., 2017), m^5^C—first characterized in tRNAs and rRNAs (Schaefer et al., 2009)—was only recently detected in mRNAs, where it modulates gene expression and development (Squires et al., 2012; Edelheit et al., 2013; David et al., 2017). In human cells, the methyltransferase NSUN2 deposits m^5^C on mRNAs (Hussain et al., 2013), whereas the adaptor protein ALYREF recognizes this mark to facilitate mRNA export (Yang et al., 2017). Similarly, in plants, m^5^C deposition alters steady-state transcript levels and influences development (Cui et al., 2017). Strikingly, the distribution and functional significance of m^5^C in filamentous fungi, especially during host infection, remain almost entirely uncharacterized

*Magnaporthe oryzae* is a well-established model for dissecting the molecular basis of fungal–plant pathogenesis. A core infection strategy involves the formation and maturation of appressoria, specialized cells that generate the mechanical force required to breach the host surface (Veneault-Fourrey et al., 2006; Deng et al., 2009; Kershaw and Talbot, 2009). The development of these infection structures is tightly linked to autophagy, a conserved intracellular recycling pathway that reallocates nutrients and maintains cellular homeostasis during host colonization (Mizushima, 2007; Nakatogawa et al., 2009).

Autophagy is driven by a suite of autophagy-related (Atg) proteins. In yeast, 36 Atg proteins have been identified, 15 of which constitute the core machinery that directs autophagosomal membrane formation (Nakatogawa, 2013). Two ubiquitin-like conjugation systems are central to this process (Suzuki et al., 2007). In the first, Atg12 is activated by the E1-like enzyme Atg7, transferred to the E2-like enzyme Atg10, and covalently linked to Atg5. The resulting Atg12–Atg5 conjugate binds to Atg16, forming a higher-order complex that functions as an E3 ligase for the second ubiquitin-like reaction, the lipidation of Atg8 with phosphatidylethanolamine (PE) (Ichimura et al., 2000; Hanada et al., 2007; Hamasaki et al., 2013). Atg8–PE associates with the expanding autophagosomal membrane and is essential for its closure and subsequent fusion with the vacuole. Therefore, the Atg12–Atg5–Atg16 complex not only catalyzes Atg8 lipidation but also determines the subcellular site at which it occurs, ensuring accurate autophagosome biogenesis (Fujita et al., 2008).

In the present study, we profiled the m^5^C RNA methylome of *M. oryzae* using methylated RNA immunoprecipitation sequencing (MeRIP-seq) and identified the methyltransferase Ncl1 as a critical positive regulator of virulence and appressorium development. Deletion of *NCL1* markedly impaired appressorium formation and maturation, phenotypes that were traced to defective autophagy. Mechanistically, we found that Ncl1-mediated m^5^C methylation stabilized *ATG5* and *ATG16* mRNAs, thereby supporting Atg8–PE production and autophagosome assembly. Post-translational regulation of Ncl1 is equally stringent: the Pmk1 mitogen-activated protein kinase (MAPK) pathway phosphorylates the enzyme, and Smt3-dependent SUMOylation further modulates its stability. Together, these findings identify an epigenetic checkpoint that links m^5^C RNA methylation to autophagy and appressorium function, providing novel targets for the management of rice blast disease.

## Results

### Identification of an m^5^C RNA methyltransferase in *M. oryzae*

**Figure 1:**
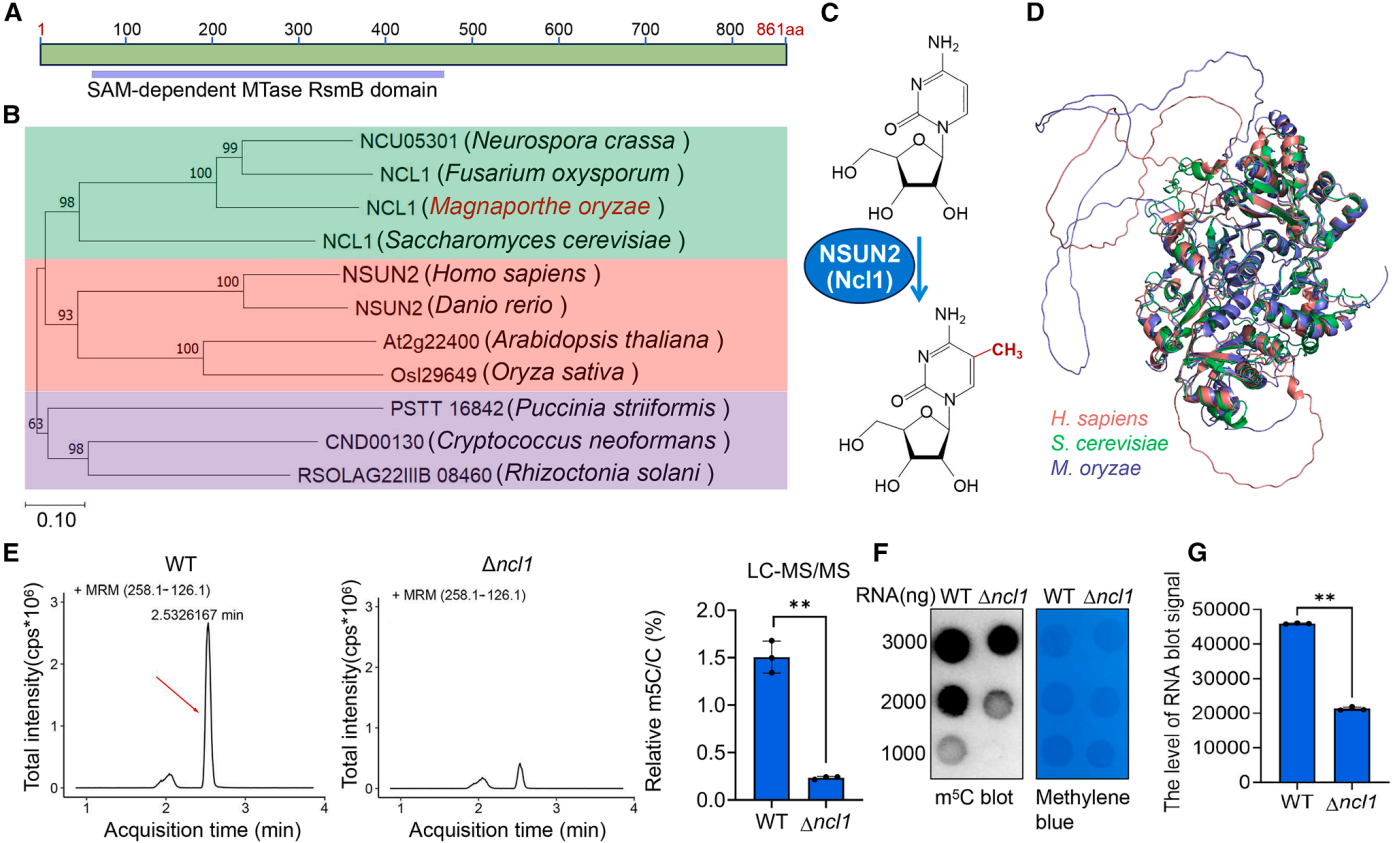
Role of Ncl1 in m^5^C RNA methylation in *Magnaporthe oryzae*. **(A)** Protein structure of *M. oryzae* Ncl1, obtained from the UniProt database (https://www.uniprot.org/). **(B)** Phylogenetic analysis. A phylogenetic tree was constructed using MEGA11 to compare *M. oryzae* Ncl1 with homologous proteins and RNA methyltransferases from different species. The tree was generated using the neighbor-joining method, and the reliability of each node was assessed using 1000 bootstrap replicates. Bootstrap values are indicated at the nodes. **(C)** NSUN2 mechanism. The enzyme catalyzes the transfer of a methyl group to the C5 position of cytosine; the methyl group is highlighted in red. **(D)** Predicted 3D structures of NSUN2 for human (*Homo sapiens*), yeast (*Saccharomyces cerevisiae*), and rice blast fungus (*M. oryzae*), illustrating structural similarities and differences. We performed structural alignment analyses of MoNCL1 with HsNSUN2 and ScNCL1 using PyMOL (version 2.3.0). The calculated root-mean-square deviation values were 1.044 Å and 1.003 Å, respectively, indicating a high degree of structural similarity in each case. A structural superimposition illustrating the alignment of MoNCL1 with HsNSUN2 and ScNCL1 is provided. **(E)** m^5^C/C ratio analysis by LC-MS/MS. The ratio of m^5^C to cytosine in wild-type (WT) and Δ*ncl1* mutant strains is shown. Error bars denote SD, and asterisks indicate statistically significant differences (∗∗*p <* 0.01). Data are means ± SD from three biological replicates. Statistical significance was determined using two-sided unpaired Student’s *t-tests* (∗∗*p* < 0.01). **(F)** RNA dot blot analysis. m^5^C levels in the WT and Δ*ncl1* mutant strains were assessed using an m^5^C-specific antibody. Total RNA was extracted, spotted onto Hybond-N+ membranes, and probed with the anti-m^5^C antibody. Detection was performed using an enhanced chemiluminescence system, with methylene blue staining as a loading control. **(G)** Quantification of RNA dot blot analysis. The intensity of the RNA blot signal (2 μg RNA per sample) was quantified using ImageJ. Data are means ± SD from three biological replicates. Statistical significance was determined using two-sided unpaired Student’s *t-tests* (∗∗*p* < 0.01).

To investigate m^5^C biology in *M. oryzae*, we searched its genome for a homolog of the human cytosine-5 RNA methyltransferase NSUN2. This analysis identified *MGG_13217* (hereafter Ncl1), an 861-residue protein with a SAM-dependent MTase RsmB domain (a hallmark of methyltransferase activity; Figure 1A). Phylogenetic analysis using MEGA11 revealed that Ncl1 is conserved across eukaryotes and clusters closely with NCU05301 from *Neurospora crassa* and the Ncl1 ortholog from *Fusarium oxysporum* (Figure 1B). Like other NSUN2 family members, Ncl1 is predicted to catalyze methylation at the C5 position of cytosine (Figure 1C). Superposition of AlphaFold-predicted structures from human, yeast, and *M. oryzae* revealed a shared catalytic fold with only minor conformational differences (Figure 1D).

To assess the cellular function of Ncl1, we generated two independent Δ*ncl1* knockout mutants that displayed identical phenotypes (Supplemental Figure 1). Liquid chromatography–tandem mass spectrometry (LC–MS/MS) quantification of total mRNA revealed a significant reduction in the m^5^C/C ratio in the mutants relative to the wild type (WT) (Figure 1E). This result was corroborated by m^5^C-specific dot blot assays, which showed a marked decrease in global m^5^C levels in Δ*ncl1* relative to the WT control (Figure 1F and 1G). Together, these data establish Ncl1 as the principal enzyme responsible for m^5^C RNA methylation in *M. oryzae*.

### *NCL1* is required for full virulence in *M. oryzae*

To investigate the role of Ncl1 in the development of *M. oryzae*, we compared the colony morphology of the Δ*ncl1* mutant, the wild type, and the complemented strain cNCL1 grown on oatmeal–tomato agar (OTA) plates at 28°C for 5 days. No significant differences were observed between the Δ*ncl1* mutant and the wild type (Supplemental Figure 2). However, the Δ*ncl1* mutant exhibited a 75% reduction in conidiation capacity compared with both the wild type and the complemented strain (Supplemental Figure 2). This suggests that *NCL1* is crucial for conidium formation but does not affect vegetative growth in *M. oryzae*.

**Figure 2:**
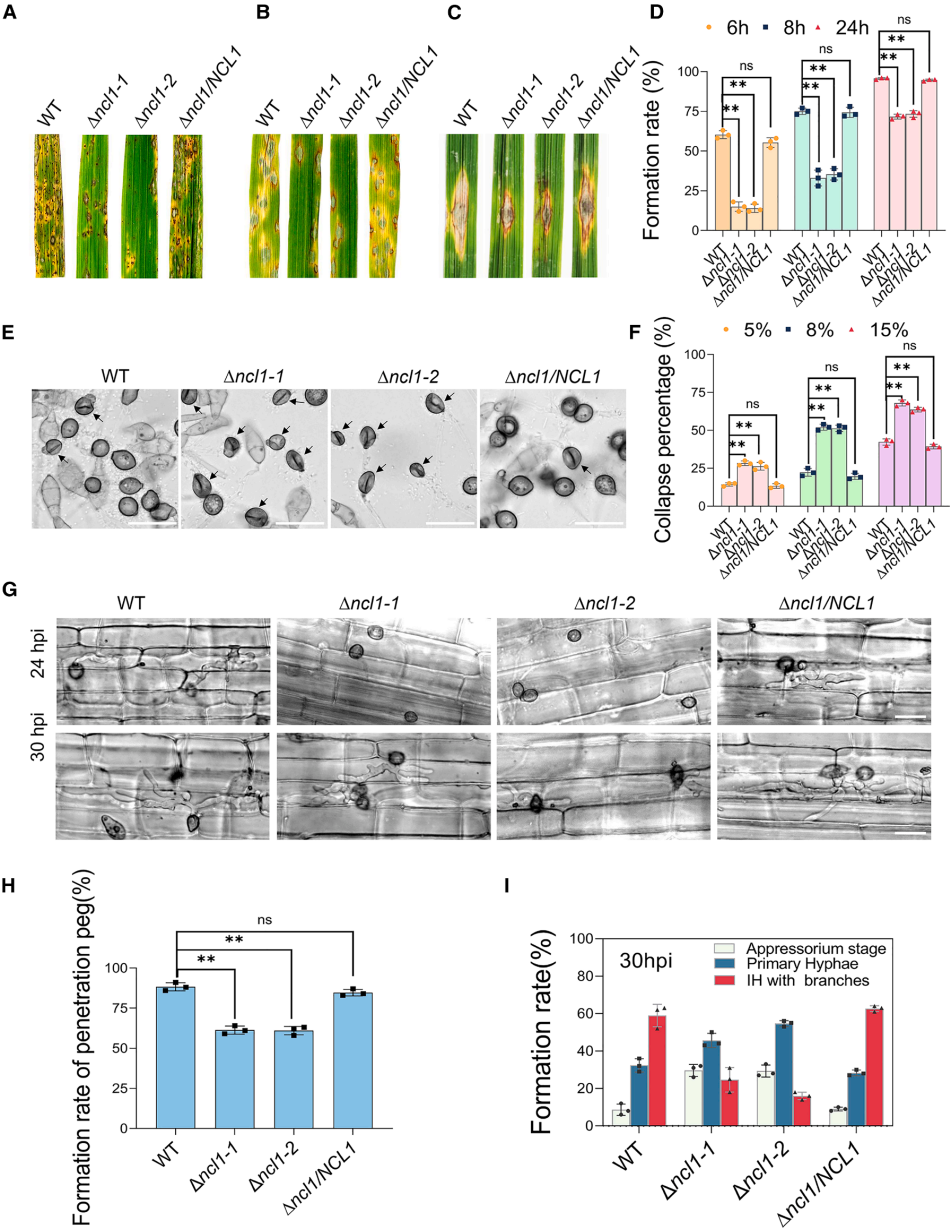
Deletion of *NCL1* leads to reduced virulence. **(A)** Rice leaves were inoculated with conidial suspensions of the wild type (WT), the Δ*ncl1* mutant, and the complemented strain. Representative leaves were photographed at 5 days post-inoculation (dpi). Experiments were repeated three times with similar results. **(B)** Barley seedlings were inoculated with conidial suspensions of the WT, the Δ*ncl1* mutant, and the complemented strain. Representative leaves were photographed at 5 dpi. Experiments were repeated three times with similar results. **(C)** Rice leaves were lightly wounded with a needle before inoculation with a mycelial block of the WT, the Δ*ncl1* mutant, or the complemented strain. Representative leaves were photographed at 4 dpi. Experiments were repeated three times with similar results. **(D)** Conidia were incubated on hydrophobic surfaces and the appressorium formation ratio was calculated at various time points (6, 8, and 24 h). Data are means ± SD from three biological replicates (*n* > 100 conidia per replicate). Statistical significance was determined by one-way ANOVA followed by Dunnett’s test (vs. WT; ∗∗*p* < 0.01; ns, not significant). **(E)** Morphological changes in appressoria treated with 15% glycerol. Arrows indicate collapsed appressoria. Scale bar, 20 μm. **(F)** The percentage of collapsed appressoria at different glycerol concentrations. Data are means ± SD from three biological replicates (*n* > 100 conidia per replicate). Statistical significance was determined by one-way ANOVA followed by Dunnett’s test (vs. WT; ∗∗*p* < 0.01; ns, not significant). **(G)** Penetration peg formation on rice leaf sheaths at 24 and 30 hpi in the WT, the Δ*ncl1* mutant, and the complemented strains. Scale bar, 20 μm. **(H)** Statistical analysis of appressorium penetration rates at 24 hpi. Data are means ± SD from three biological replicates (*n* > 100 appressoria per replicate). Statistical significance was determined by one-way ANOVA followed by Dunnett’s test (vs. WT; ∗∗*p* < 0.01; ns, not significant). **(I)** Statistical analysis of different types of infection hyphae at 30 hpi. Data are means ± SD from three biological replicates (*n* > 100 appressoria per replicate). Statistical significance was determined by one-way ANOVA followed by Dunnett’s test (vs. WT; ∗∗*p* < 0.01; ns, not significant).

We subsequently evaluated the effect of *NCL1* deletion on *M. oryzae* pathogenicity by inoculating conidial suspensions onto susceptible rice seedlings and barley leaves. The wild type and complemented strains produced abundant spreading lesions, whereas the Δ*ncl1* mutant resulted in significantly fewer lesions (Figure 2A and 2B). This indicates a substantial reduction in pathogenicity toward both rice and barley in the Δ*ncl1* mutant. Furthermore, when mycelial agar plugs were placed onto wounded rice leaves, the Δ*ncl1* mutant showed limited lesion expansion (Figure 2C). These findings confirm that Ncl1 is essential for full virulence in *M. oryzae*.

### *NCL1* disruption affects appressorium formation and maturation

To investigate why Δ*ncl1* is defective in host penetration, we monitored appressorium development on hydrophobic coverslips. At 6 and 8 h post inoculation (hpi), the Δ*ncl1* mutant showed a significantly lower appressorium formation rate than the WT, indicating delayed differentiation (Figure 2D). By 24 hpi, more than 90% of wild-type conidia had formed appressoria, whereas only ∼75% of Δ*ncl1* conidia had done so, confirming that Ncl1 is required for efficient appressorium initiation. Cytorrhysis assays further showed that glycerol-treated Δ*ncl1* appressoria collapsed more readily than those of the WT or complemented strains (Figure 2E and 2F), indicating that Ncl1 also supports cell wall rigidity and turgor-driven maturation.

We next traced the infection process in living host tissue. In rice leaf sheaths, the wild-type and complemented strains produced penetration pegs from >85% of appressoria by 24 hpi, whereas the Δ*ncl1* mutant achieved penetration in only ∼60% of cases (Figure 2G and 2H). By 30 hpi, branched invasive hyphae were evident in >55% of wild-type penetration sites but in <30% of Δ*ncl1* sites (Figure 2G and 2I). Similar results were obtained in barley epidermis (Supplemental Figure 3). Collectively, these data demonstrate that Ncl1 is essential not only for appressorium-mediated penetration but also for the subsequent expansion of invasive hyphae in *M. oryzae*.

### Transcriptome-wide m^5^C mapping by MeRIP-seq in *M. oryzae*

**Figure 3:**
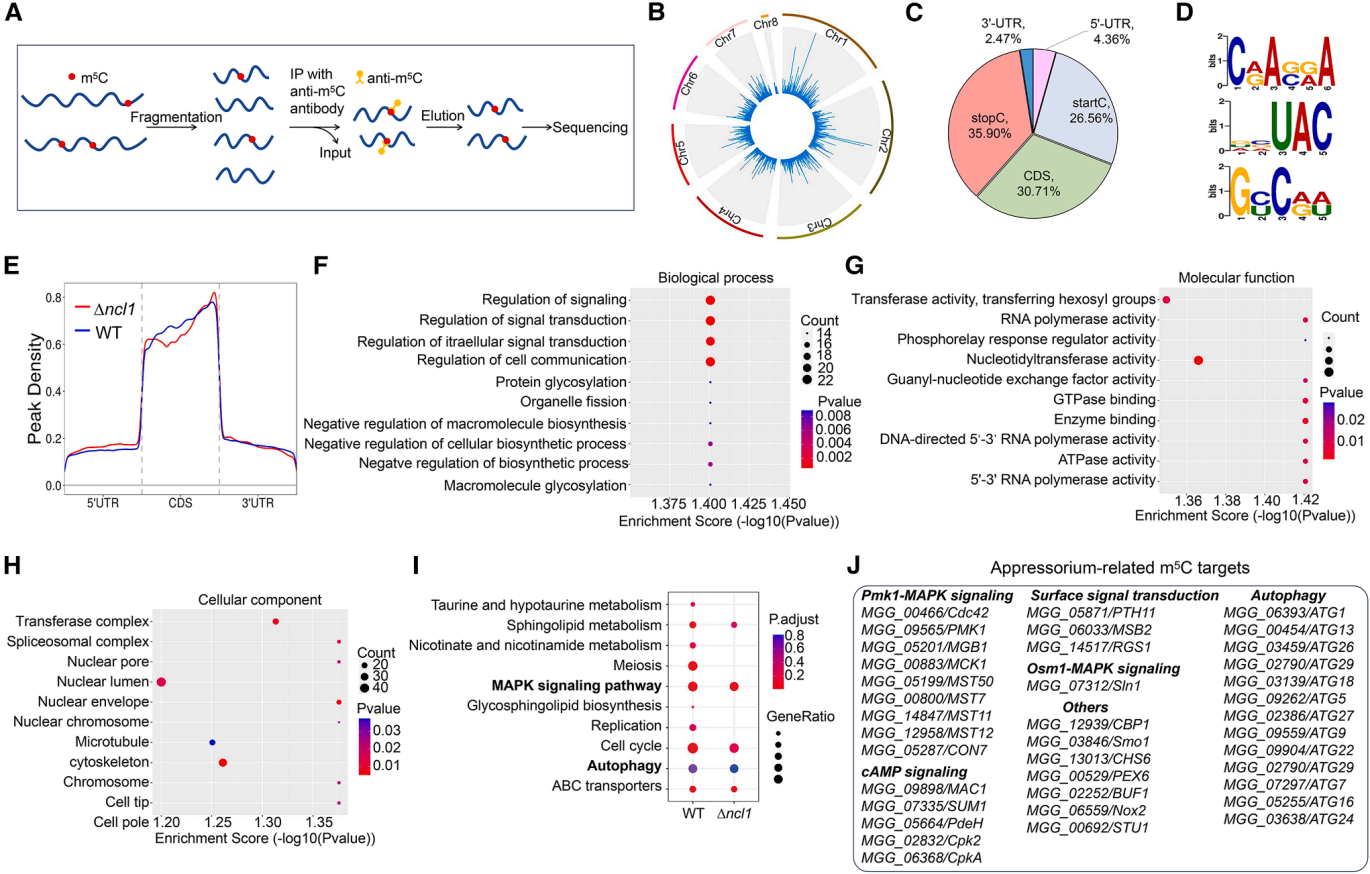
Comprehensive profiling of Ncl1-mediated m^5^C methylation in *M. oryzae* transcripts. **(A)** Illustration of the mRNA m^5^C sequencing process. **(B)** Chromosomal mapping and visualization of m^5^C methylation in *M. oryzae*. **(C)** Distribution of m^5^C along *M. oryzae* mRNA transcripts. **(D)** The m^5^C motifs with the lowest E values for enhanced sensitivity and specificity in transcriptomic analysis. **(E)** Relative m^5^C levels across mRNA regions in the WT and the Δ*ncl1* mutant. **(F)** GO biological process classification of m^5^C-modified mRNAs in Δ*ncl1* versus WT. **(G)** GO molecular function classification of m^5^C-modified mRNAs in Δ*ncl1* versus WT. **(H)** GO cellular component classification of m^5^C-modified mRNAs in Δ*ncl1* versus WT. **(I)** Kyoto Encyclopedia of Genes and Genomes pathway analysis highlighting the metabolic pathways most affected by downregulation of m^5^C-modified mRNAs in Δ*ncl1* versus WT. **(J)** m^5^C-modified transcripts related to appressorium development with significantly reduced methylation in the Δ*ncl1* mutant.

To investigate the biological significance and underlying mechanisms of m^5^C methylation in *M. oryzae*, we performed m^5^C sequencing in the WT and Δ*ncl1* strains. m^5^C MeRIP-seq was conducted using RNA extracted from mycelia of the wild-type strain P131 and the Δ*ncl1* mutant. The RNA was fragmented into approximately 100-nucleotide (nt) segments (input) and subjected to immunoprecipitation with an anti-m^5^C antibody (Figure 3A). Libraries were prepared from both the immunoprecipitated (IP) and input control RNA samples, with two biological replicates showing high Pearson correlation coefficients. After removing reads aligned to annotated rRNA sequences, unique reads from the IP and input samples were mapped to the *M. oryzae* genome. We observed a significant reduction in m^5^C enrichment at 9014 peaks corresponding to mRNAs from 5678 genes in the Δ*ncl1* mutant compared with the wild-type strain (Supplemental Table 1).

We used Circos (Krzywinski et al., 2009) to analyze the chromosomal distribution of m^5^C peaks and observed variations in both the number and distribution of peaks, with chromosome 1 showing the most pronounced differences (Figure 3B). We further characterized the enrichment pattern of m^5^C sites within mRNA transcripts. Peaks were classified into the following categories based on their positions within transcripts: 5′ untranslated region (UTR) (4.36%), start codon region (26.56%), coding sequence (CDS) (30.71%), stop codon region (35.90%), and 3′ UTR (2.47%). Notably, m^5^C sites were significantly enriched in the start codon, CDS, and stop codon regions (Figure 3C). Motif analysis identified CRAYRA (R = A/G; Y = C/G) as the most prevalent and best-supported motif, representing the most likely conserved methylation site (E value = 4.6e^−59^). Other significant motifs included KVUAC (K = G/U/A; V = C/G/A; E = 1.3e^−52^) and GMCRW (M = C/U; R = A/G; W = A/U; E = 3.8e^−43^) (Figure 3D). Compared with the wild type, the Δ*ncl1* mutant showed significantly fewer m^5^C peaks within CDS regions (Figure 3E).

### Functional categorization of m^5^C-methylated gene transcripts in *M. oryzae*

To investigate the functional landscape of m^5^C-methylated transcripts in *M. oryzae*, we conducted a comprehensive Gene Ontology (GO) analysis. Biological process terms showed pronounced enrichment for signaling regulation, signal transduction, and intracellular signal transduction; cell communication and protein glycosylation were also highly represented. In addition, m^5^C-marked transcripts were significantly associated with negative regulation of macromolecule biosynthesis, cellular biosynthetic processes, and general biosynthetic pathways (Figure 3F), implying that m^5^C methylation fine-tunes anabolic activity. These transcripts were also associated with cellular components such as the transferase complex, spliceosome, nuclear pores, nuclear lumen, nuclear envelope, chromosomes, and the microtubule cytoskeleton (Figure 3H). These results indicate that m^5^C-modified mRNAs are biased toward nuclear and cytoskeletal components. Molecular function analysis revealed over-representation of hexosyltransferase activity, RNA polymerase activity, and phosphorelay response regulator activity. Nucleotidyltransferase activity, guanyl nucleotide exchange factor activity, and GTPase binding were also enriched (Figure 3G), indicating involvement in diverse molecular processes. Enzyme binding, DNA-directed 5′→3′ RNA polymerase activity, ATPase activity, and 5′→3′ RNA polymerase activity were also identified, highlighting the broad functional repertoire of m^5^C-methylated gene products. Kyoto Encyclopedia of Genes and Genomes pathway enrichment analysis of genes with reduced m^5^C peaks revealed strong associations with meiosis, the MAPK signaling cascade, DNA replication, cell cycle control, autophagy, and the metabolism of taurine/hypotaurine, sphingolipids, and nicotinate/nicotinamide (Figure 3I). Collectively, these data underscore the central regulatory role of m^5^C methylation in the core cellular processes of *M. oryzae*.

Functional integration revealed that Ncl1-dependent m^5^C marks define a coherent regulatory network governing appressorium development and pathogenicity. In the context of signal transduction, m^5^C stabilizes transcripts encoding Pmk1–MAPK components (*MGG_00466/Cdc42*, *MGG_00883/MCK1*, *MGG_00800/MST7*, *MGG_14847/MST11*, *MGG_09565/PMK1*), cyclic AMP (cAMP) pathway genes (*MGG_09898/MAC1*, *MGG_05664/PdeH*, *MGG_02832/Cpk2*, *MGG_06368/CpkA*), surface-sensing genes (*MGG_05871/PTH11*, *MGG_06033/MSB2*, *MGG_12939/CBP1*), and the Osm1–MAPK regulator *MGG_07312/SLN1*, thereby initiating appressorium formation. In macromolecular biosynthesis, m^5^C protects mRNAs encoding autophagy factors (*ATG1*, *ATG5*, *ATG7*, *ATG16*), cell wall synthesis enzymes (*MGG_13013/CHS6*, *MGG_02252/BUF1*), nutrient utilization proteins (*MGG_00529/PEX6*, *MGG_00692/STU1*), and membrane homeostasis genes (*MGG_03846/Smo1*, *MGG_06559/NOX2*), thereby driving appressorium maturation and sustaining glycerol-dependent turgor and cell wall integrity (Figure 3J). This network positions Ncl1-mediated m^5^C methylation as a central epigenetic mechanism that couples post-transcriptional modification to the molecular processes underlying appressorium function and fungal virulence.

### *NCL1* deletion alters the pattern of gene expression in *M. oryzae*

**Figure 4:**
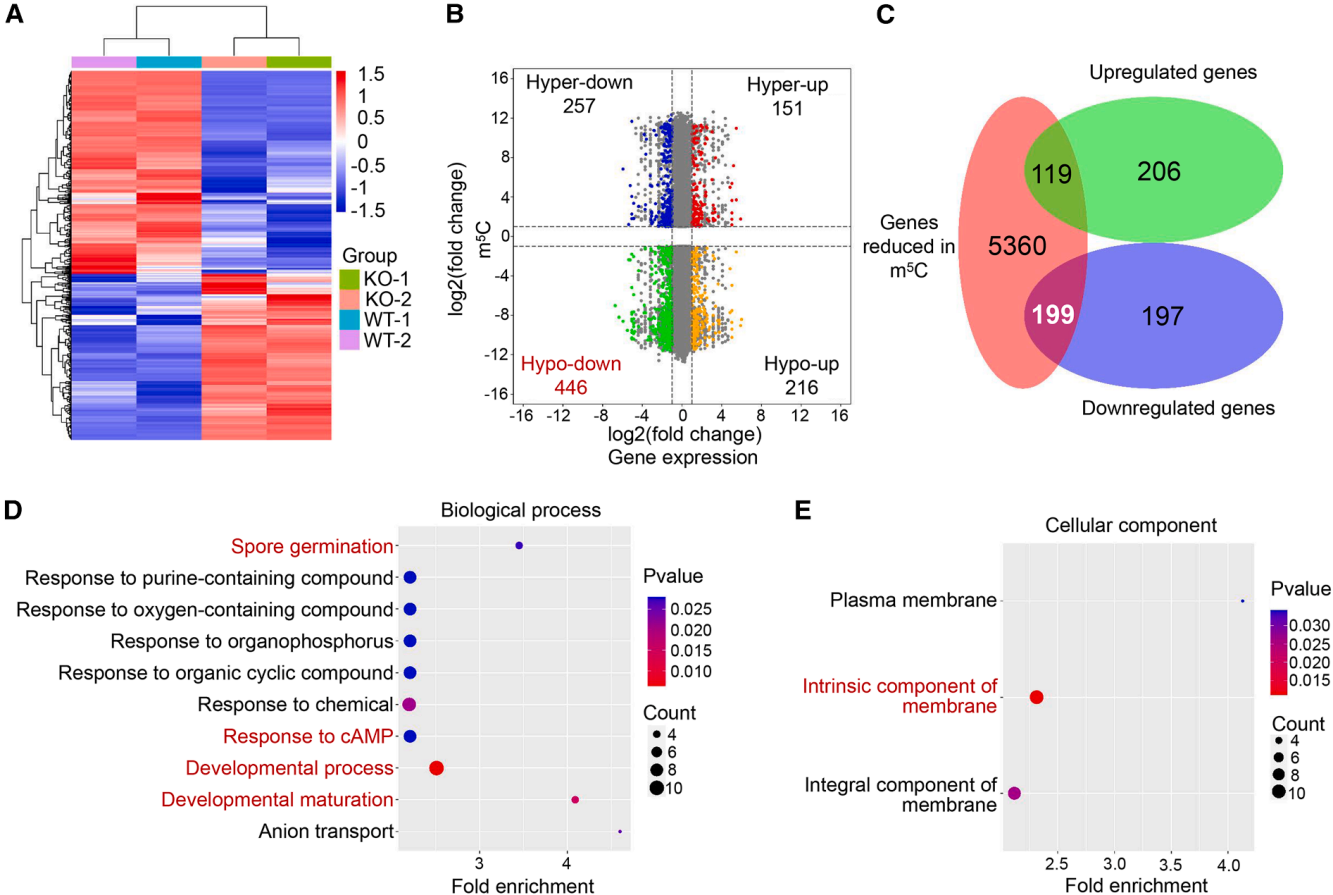
Comprehensive analysis of m^5^C-seq and RNA-seq data reveals transcripts influenced by Ncl1. **(A)** Heatmap highlighting DEGs in WT (WT1 and WT2) and Δ*ncl1* mutant strains (knockout [KO]1 and KO2). **(B)** Four-quadrant plots showing the correlation between changes in m^5^C methylation and mRNA levels. **(C)** Venn diagram showing the overlap between genes with reduced m^5^C methylation, upregulated genes, and downregulated genes in the Δ*ncl1* mutant. **(D)** GO biological process classification of downregulated mRNAs with decreased m^5^C levels in the Δ*ncl1* mutant compared with WT. **(E)** GO cellular component classification of downregulated mRNAs with decreased m^5^C levels in the Δ*ncl1* mutant compared with WT.

To comprehensively assess how m^5^C methylation in *M. oryzae* influences gene expression at the transcriptional level, we performed a comparative transcriptome analysis of the wild type and the Δ*ncl1* mutant. Unsupervised clustering of RNA sequencing (RNA-seq) data revealed a clear divergence in global transcriptomic profiles between the two strains (Figure 4A), indicating that m^5^C methylation exerts broad and significant effects on gene regulation. *NCL1* deletion reshaped the *M. oryzae* transcriptome, resulting in 325 upregulated and 396 downregulated differentially expressed genes (DEGs) (Supplemental Table 2), many of which are directly linked to pathogenicity.

Downregulated DEGs included key virulence-associated genes such as *MGG_05871/PTH11* (required for appressorium initiation), *MGG_12939/CBP1* (involved in host adhesion), *MGG_00461/MoAPX1* (responsible for reactive oxygen species scavenging), *MGG_06064/CHS7* (contributing to cell wall rigidity), *MGG_01173/MHP1* (involved in nutrient uptake), and *MGG_02884/MoFLP1* (involved in nuclear division). The reduced expression of these genes is consistent with delayed or impaired penetration ability. In contrast, upregulated DEGs included several temporal negative regulators such as *MGG_10705/Rbf1* (a suppressor of cell wall-degrading enzymes), *MGG_15370/AVR-Pita* (which helps avoid premature host recognition), and *MGG_04795/BAS1* and *MGG_09693/BAS2* (both of which repress biotrophy-associated genes) (Supplemental Table 2). Their aberrant upregulation in the Δ*ncl1* mutant likely increases pre-penetration repression. Together, these DEGs indicate that Ncl1 acts as a temporal and functional regulatory hub: m^5^C methylation stabilizes transcripts essential for early infection and coordinates stage-specific gene expression to ensure that post-penetration programs are activated only after appressorial maturation. Loss of Ncl1 disrupts this balance, resulting in dysregulated development and attenuated virulence.

To validate the RNA-seq data, we randomly selected six DEGs (three upregulated and three downregulated) and confirmed their expression patterns using reverse transcription quantitative PCR (RT–qPCR). All six transcripts exhibited changes consistent with the sequencing results (Supplemental Figure 4C and 4D), confirming the reliability of our transcriptomic dataset. Next, we integrated MeRIP-seq and RNA-seq data to classify differentially methylated m^5^C peaks into four categories based on the associated changes in mRNA abundance. We identified 408 hypermethylated m^5^C peaks corresponding to genes that were either significantly upregulated (151 genes; hyper-up) or downregulated (257 genes; hyper-down). In contrast, 662 hypomethylated m^5^C peaks were associated with genes that were either upregulated (216 genes; hypo-up) or downregulated (446 genes; hypo-down) (Figure 4B). Among the 5678 hypomethylated mRNAs detected in the Δ*ncl1* mutant, 119 were transcriptionally upregulated and 199 were downregulated (Figure 4C). These findings indicate that loss of m^5^C methylation is associated with both increased and decreased transcript abundance, suggesting context-dependent effects on mRNA stability or secondary regulatory feedback. Overall, our data demonstrate that disruption of Ncl1 leads to a global reduction in m^5^C methylation and significant alterations in gene expression, underscoring the critical role of Ncl1 and m^5^C methylation in transcriptional regulation in *M. oryzae*.

GO enrichment analysis of downregulated transcripts in the Δ*ncl1* mutant revealed significant over-representation of biological processes, including spore germination; responses to purine-containing, oxygen-containing, organophosphorus, and organic cyclic compounds; responses to general chemical stimuli and cAMP; developmental processes; and anion transport (Figure 4D). In the cellular component category, there was notable enrichment in plasma membrane and membrane components, including integral membrane proteins (Figure 4E). These results highlight the broad range of biological processes and cellular components affected by the downregulation of gene expression in the Δ*ncl1* mutant, further emphasizing the importance of Ncl1 in maintaining proper transcriptional control in *M. oryzae*.

### Ncl1-mediated m^5^C methylation ensures *ATG* gene expression during appressorium formation

Autophagy, an intracellular degradation pathway that is highly conserved from yeast to mammals (Feng et al., 2014; Ohsumi, 2014), is essential for the morphogenesis and maturation of appressoria in *M*. *oryzae*, the fungus responsible for rice blast disease (Veneault-Fourrey et al., 2006; Saunders et al., 2010). Our study demonstrates that the m^5^C methyltransferase Ncl1 is a key regulator of both appressorium formation and maturation in *M. oryzae* (Figure 2). We therefore investigated whether Ncl1 exerts these effects through modulation of autophagy.

**Figure 5:**
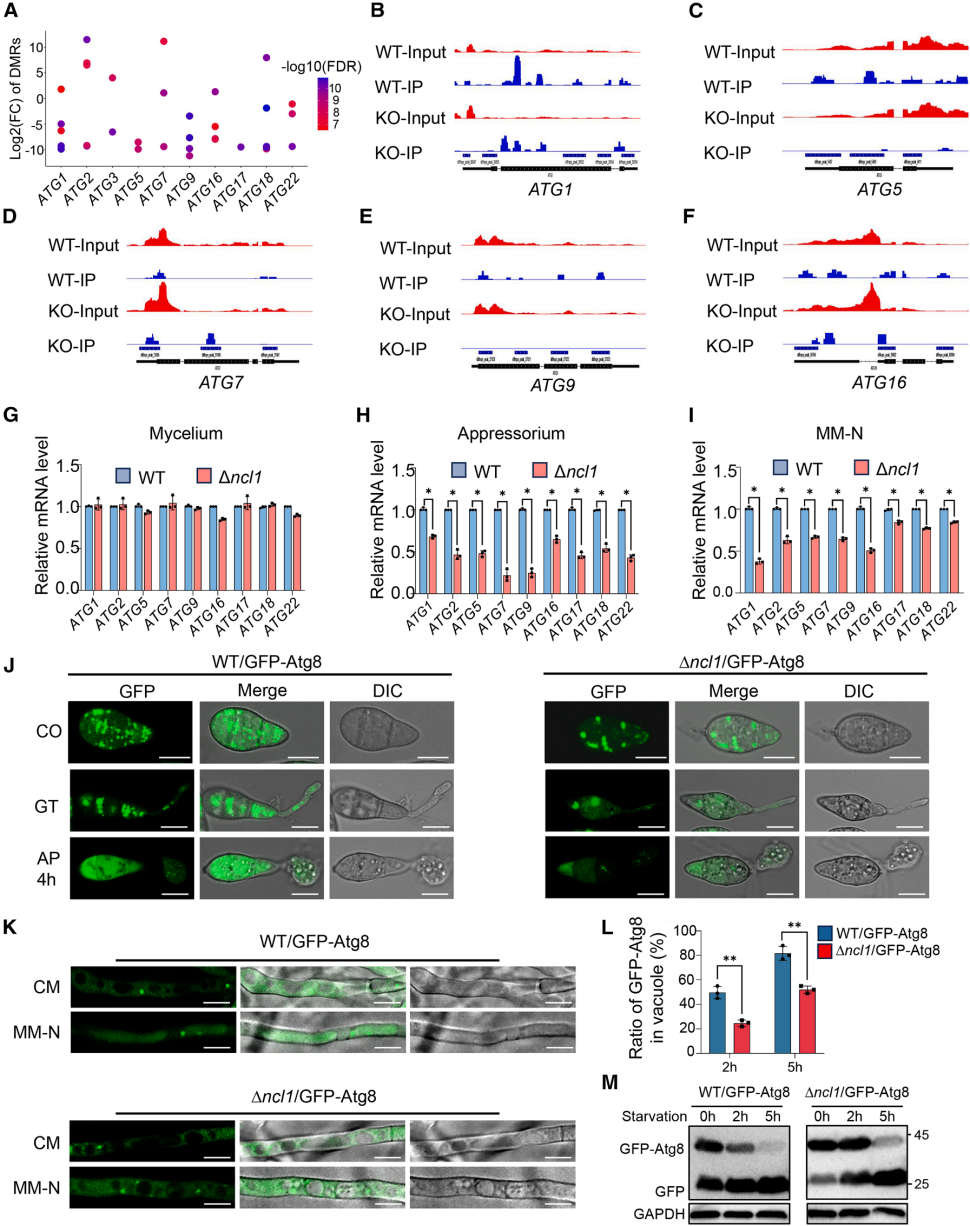
Ncl1-mediated m^5^C methylation influences *ATG* gene transcription during appressorium formation. **(A)** Plot showing fold changes in m^5^C methylation for autophagy-related gene transcripts affected by Ncl1. Color coding indicates the significance of differences between the WT and the Δ*ncl1* mutant (KO). **(B–F)** Integrative Genomics Viewer plots illustrating m^5^C peak patterns for transcripts of autophagy-related genes in *M. oryzae*. **(G)** Real-time RT–qPCR analysis of *ATG* mRNA levels in vegetative hyphae cultured in CM for 36 h. Data are means ± SE from three biological replicates. Statistical significance was determined using two-sided unpaired Student’s *t-tests* (∗*p* < 0.05). **(H)** RT–qPCR analysis of *ATG* mRNA levels in appressoria grown on a hydrophobic membrane for 6 h. Data are means ± SE from three biological replicates. Statistical significance was determined using two-sided unpaired Student’s *t-tests* (∗*p* < 0.05). **(I)** RT–qPCR analysis of *ATG* mRNA levels in vegetative hyphae cultured in CM for 36 h and then transferred to nitrogen-deficient medium (MM-N) for 6 h. Data are means ± SE from three biological replicates. Statistical significance was determined using two-sided unpaired Student’s *t-tests* (∗*p* < 0.05). **(J)** Subcellular localization of GFP-Atg8 in conidia, germ tubes, and appressoria at 4 h post germination. AP, appressorium; CO, conidium; GT, germ tube. Images were captured by fluorescence microscopy. Scale bar, 10 μm. Experiments were repeated three times with similar results. **(K)** Subcellular localization of GFP-Atg8 under nutrient-deficient conditions. Strains expressing GFP-Atg8 were cultured in CM for 36 h and then transferred to MM-N for 5 h. Images were captured by fluorescence microscopy. Scale bar, 10 μm. Experiments were repeated three times with similar results. **(L)** Quantification of autophagy intensity based on vacuolar translocation of GFP-Atg8 (*n* > 100). Data are means ± SD from three biological replicates (*n* > 100 hyphae per replicate). Statistical significance was determined using two-sided unpaired Student’s *t*-tests (∗∗*p* < 0.01). **(M)** Immunoblot analysis using an anti-GFP antibody. An anti-GAPDH antibody was used as a loading control. Experiments were repeated three times with similar results.

Thirty-two autophagy-related (*ATG*) genes are present in the *M. oryzae* genome, many of which are closely linked to appressorium development and pathogenicity (Kershaw and Talbot, 2009). MeRIP-seq analysis revealed that Ncl1 influences m^5^C methylation of 11 *ATG* transcripts: *ATG1*, *ATG2*, *ATG3*, *ATG5*, *ATG7*, *ATG9*, *ATG11*, *ATG16*, *ATG17*, *ATG18*, and *ATG22* (Figure 5A–5F; Supplemental Table 3). Among these, the m^5^C levels of eight *ATG* mRNAs (all except *ATG2*, *ATG3*, and *ATG11*) were significantly reduced in the Δ*ncl1* mutant (Supplemental Table 3).

To determine whether these methylation changes affect gene expression, we measured the transcript abundance of the 11 *ATG* genes in three contexts: (1) vegetative hyphae grown in complete medium (CM) for 36 h, (2) vegetative hyphae transferred to nitrogen-deficient medium (MM-N) for 6 h, and (3) appressoria formed on hydrophobic membranes for 6 h. Real-time RT–qPCR showed that disruption of *NCL1* had no discernible effect on *ATG* gene expression during vegetative growth in CM (Figure 5G). In contrast, under nitrogen starvation and during appressorium development, most *ATG* transcripts were markedly reduced in the Δ*ncl1* mutant relative to the wild type and complemented strains (Figures 5H and 5I). These results indicate that Ncl1-mediated m^5^C methylation is not required for basal expression of autophagy genes but is critical for their upregulation during autophagy induction. Overall, our findings suggest that m^5^C methylation stabilizes *ATG* mRNAs, thereby ensuring sufficient autophagic flux for appressorium maturation and host infection.

### Ncl1 mediates autophagy in an m^5^C-dependent manner

Atg8 is a ubiquitin-like protein that serves as a hallmark of autophagosomes in yeast (Kirisako et al., 1999). To evaluate the effect of *NCL1* disruption on autophagy, we introduced a GFP-Atg8 fusion construct into both the wild type and the Δ*ncl1* mutant. The presence and intracellular distribution of GFP-Atg8-labeled autophagosomes were monitored by fluorescence microscopy. During appressorium formation, we observed a marked reduction in the number of GFP-Atg8 puncta in the Δ*ncl1* background relative to the WT (Figure 5J).

To quantify autophagic flux, hyphae were subjected to nitrogen starvation for 5 h. Under these conditions, approximately 80% of WT autophagosomes reached and fused with the vacuole, whereas approximately 50% did so in Δ*ncl1* (Figure 5K and 5L), indicating impaired delivery or degradation of autophagic bodies. Western blot analysis of total protein extracts corroborated the microscopy data. In the WT, the GFP-Atg8 signal declined progressively during starvation, reflecting normal vacuolar degradation of the fusion protein. In contrast, GFP-Atg8 levels decreased only marginally in the Δ*ncl1* mutant, demonstrating a significantly slower turnover rate (Figure 5M; Supplemental Figure 12H). Overall, these results establish Ncl1 as a critical facilitator of autophagy in *M. oryzae*, a function that underpins proper appressorium development and full pathogenic potential.

### Ncl1-mediated m^5^C methylation promotes *ATG* mRNA stability and Atg8lipidation

**Figure 6:**
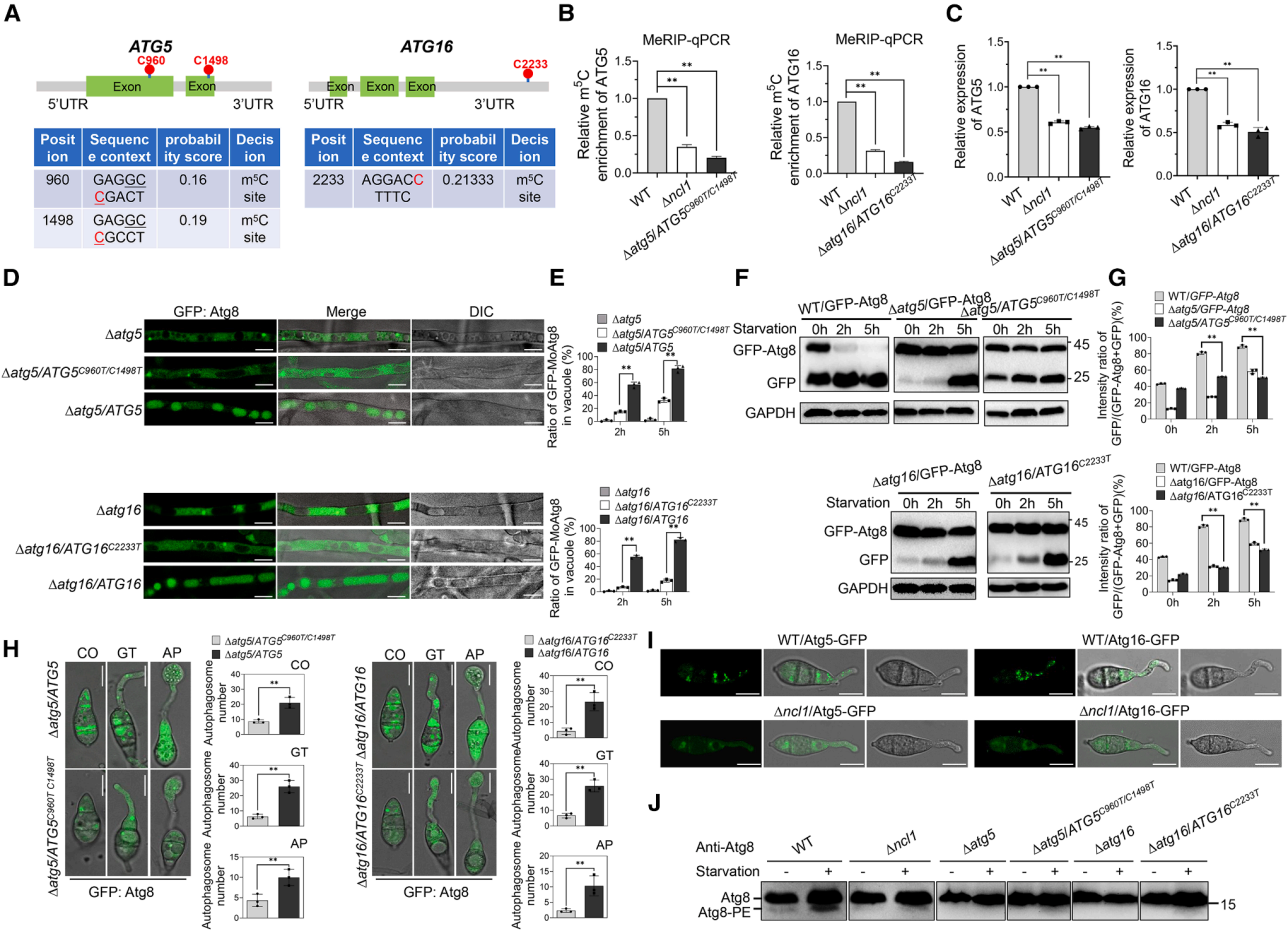
Ncl1 ensures autophagy in an m^5^C-dependent manner. **(A)** Prediction of potential m^5^C RNA methylation sites using RNAnut (http://www.rnanut.net/rnam5Cfinder/). The C-to-T mutations introduced at the m^5^C sites in *ATG5* (GCC to GCT) did not result in amino acid changes, and the C-to-T mutation introduced at the m^5^C site in ATG16 is located in the 3′ untranslated region (UTR); therefore, these mutations do not affect amino acid identity. **(B)** Methylated RNA immunoprecipitation–qPCR (MeRIP–qPCR) analysis of m^5^C levels in *ATG5* and *ATG16* in *M. oryzae*. Appressorium samples from WT and the Δ*ncl1* mutant were subjected to m^5^C-IP using an anti-m^5^C antibody. MeRIP–qPCR was subsequently performed using the IP products as templates. Data are means ± SE from three biological replicates. Statistical significance was determined using one-way ANOVA followed by Tukey’s honestly significant difference (HSD) test (∗∗*p* < 0.01). **(C)** Real-time RT–qPCR analysis of *ATG5* and *ATG16* mRNA levels. Data are means ± SE from three biological replicates. Statistical significance was determined using one-way ANOVA followed by Tukey’s HSD test (∗∗*p* < 0.01). **(D)** Subcellular localization of Atg5 and Atg16 m^5^C site mutants under nutrient-deficient conditions. Strains expressing GFP-Atg8 were cultured in CM for 36 h and then transferred to MM-N for 5 h. Images were captured by fluorescence microscopy. Scale bar, 10 μm. Experiments were repeated three times with similar results. **(E)** Quantification of autophagy intensity based on vacuolar translocation of GFP fusion proteins as described in **(D)** (*n* > 100). Data are means ± SD from three biological replicates. Statistical significance was determined using one-way ANOVA followed by Tukey’s HSD test (∗∗*p* < 0.01). **(F)** Immunoblot analysis using an anti-GFP antibody. Strains expressing GFP-Atg8 were cultured in CM for 36 h and then transferred to MM-N for 2 or 5 h. An anti-GAPDH antibody was used as a loading control. Experiments were repeated three times with similar results. **(G)** Intensity ratio of GFP/(GFP-Atg8 + GFP) in different strains as described in **(F)**. Data are means ± SD from three biological replicates. Statistical significance was determined using one-way ANOVA followed by Tukey’s HSD test (∗∗*p* < 0.01). **(H)** Subcellular localization of Atg5 and Atg16 m^5^C site mutants during appressorium formation. AP, appressorium; CO, conidium; GT, germ tube. Images were captured by fluorescence microscopy. Scale bar, 10 μm. **(I)** Subcellular localization of Atg5-GFP and Atg16-GFP in WT and the Δ*ncl1* mutant during appressorium formation. Images were captured by fluorescence microscopy. Scale bar, 10 μm. **(J)** Atg8–PE levels in different strains were analyzed by urea–SDS–PAGE containing 6 M urea and detected using an anti-Atg8 antibody.

Guided by the m^5^C-seq and transcriptome data, we hypothesized that Ncl1 governs autophagy by depositing m^5^C marks that stabilize *ATG* mRNAs. In the Δ*ncl1* background, we detected reduced m^5^C levels in eight *ATG* transcripts and, concordantly, a significant decrease in the expression of five of these genes—*ATG1*, *ATG5*, *ATG7*, *ATG9*, and *ATG16*—under nitrogen starvation or appressorium-inducing conditions. Because Atg5 and Atg16 are required for the assembly and localization of Atg8–PE, a lipidated form of Atg8 that drives autophagosome expansion and closure (Suzuki et al., 2001; Nakatogawa et al., 2007; Xie et al., 2008), we focused on identifying the m^5^C sites. By integrating our m^5^C-seq profiles with predictions generated by the online tool RNAm^5^CFinder (http://www.rnanut.net/rnam5Cfinder/), we identified two high-confidence m^5^C sites in *ATG5* and one in *ATG16* (Figure 6A). We then introduced C-to-T point mutations—C960T and C1498T in *ATG5*, and C2233T in *ATG16*—at these positions, while ensuring that coding capacity remained unchanged. The two *ATG5* mutations lie within synonymous codons (GCC→GCT), and the *ATG16* mutation resides in the 3′ UTR; therefore, any phenotypic consequences can be attributed solely to the loss of methylation and the resulting mRNA destabilization rather than to changes in protein sequence.

MeRIP–qPCR confirmed a pronounced reduction in the m^5^C levels of both *ATG5* and *ATG16* transcripts in the Δ*ncl1* mutant and the corresponding site-directed mutants (Δ*atg5/ATG5*^*C960T/C1498T*^ and Δ*atg16/ATG16*^*C2233T*^) relative to the wild-type control (Figure 6B), establishing that Ncl1 deposits m^5^C at C960 and C1498 in *ATG5* mRNA and at C2233 in *ATG16* mRNA. RT–PCR subsequently showed that steady-state levels of *ATG5* and *ATG16* mRNAs were significantly reduced in both the Δ*ncl1* strain and the methylation-site mutants (Figure 6C), indicating increased transcript turnover.

To test this directly, we performed an actinomycin D transcriptional inhibition assay. Vegetative hyphae were starved for nitrogen for 2 h, transcription was blocked with 10 μg ml^−1^ actinomycin D, and samples were collected at 0, 30, 60, 90, 120, and 240 min. RT–qPCR analysis revealed that *ATG5* and *ATG16* transcripts decayed markedly faster in Δ*ncl1* than in the wild type or in the corresponding C-to-T mutants (Supplemental Figure 4A and 4B). These results demonstrate that Ncl1-dependent m^5^C methylation stabilizes *ATG5* and *ATG16* mRNAs, thereby ensuring their translation during periods of high autophagic demand.

To assess the functional consequences of this mRNA instability, we introduced GFP-Atg8 into the Δ*atg5*, Δ*atg16*, and methylation-site mutants and their respective complemented strains. After 5 h of nitrogen starvation in MM-N, the majority of GFP-Atg8 puncta were localized to the vacuole in the complemented controls, whereas the number of autophagosomes was markedly reduced in the methylation-site mutants (Figure 6D and 6E). As expected, virtually no vacuolar delivery was observed in the Δ*atg5* or Δ*atg16* null mutants. Western blot analysis confirmed these observations: the free GFP band, which indicates vacuolar degradation of GFP-Atg8 showed greatly reduced intensity in the methylation-site mutants (Figure 6F and 6G). A similar defect in vacuolar delivery was observed during appressorium formation (Figure 6H). Collectively, these data demonstrate that Ncl1 maintains autophagic flux in an m^5^C-dependent manner.

The pre-autophagosomal structure (PAS) is the initial nucleation site at which Atg proteins, including Atg5 and Atg16, assemble into puncta (Suzuki et al., 2001). In the Δ*ncl1* mutant, Atg5-GFP and Atg16-GFP remained largely diffuse throughout the cytoplasm during appressorium development, with only rare puncta detected (Figure 6I); this indicates that Ncl1 activity is required for efficient PAS assembly, most likely through its influence on Atg8 lipidation. To quantify Atg8–PE levels, we used an anti-Atg8 antibody that recognizes both the cytosolic (Atg8) and lipidated (Atg8–PE) forms. After 4 h of nitrogen starvation in MM-N, the Δ*ncl1* mutant contained significantly less Atg8–PE than the WT. Similarly, the Δ*atg5*, Δ*atg16*, and methylation-site mutants exhibited markedly reduced Atg8–PE levels under starvation conditions (Figure 6J; Supplemental Figure 12I). This confirms that m^5^C-dependent stabilization of ATG5 and *ATG16* mRNAs is essential for normal Atg8 lipidation. In summary, our findings establish that *ATG5* and *ATG16* mRNAs are direct targets of Ncl1-mediated m^5^C methylation, that this modification prolongs their half-life, and that their sustained expression is required to maintain physiological levels of Atg8–PE, proper PAS organization, and efficient autophagic flux in *M. oryzae*.

### The m^5^C sites in *ATG5* and *ATG16* mRNAs are important for *M. oryzae* virulence

To evaluate the biological significance of the m^5^C sites within *ATG5* and *ATG16* transcripts, we compared the growth rate, conidiation capacity, and pathogenicity of the wild type, Δ*atg5*, Δ*atg16*, the corresponding m^5^C-site mutants Δ*atg5/ATG5*^*C960T/C1498T*^ and Δ*atg16/ATG16*^*C2233T*^, and their fully complemented derivatives. Radial growth on OTA plates was indistinguishable among all genotypes (Supplemental Figure 5), whereas sporulation differed markedly: the methylation-site mutants produced significantly fewer conidia than the wild type and complemented strains (Supplemental Figure 6A and 6B).

In pathogenicity assays on barley and rice, the Δ*atg5/ATG5*^*C960T/C1498T*^ and Δ*atg16/ATG16*^*C2233T*^ strains generated noticeably smaller and less numerous lesions than the wild type and complemented controls (Supplemental Figure 7A and 7B). Cytorrhysis assays revealed that appressoria formed by the methylation-site mutants collapsed more readily under glycerol treatment than those of the wild type and complemented strains (Supplemental Figure 7C and 7D), indicating impaired maturation.

Penetration assays on rice leaf sheaths showed that mutant appressoria formed penetration pegs at greatly reduced frequencies (Supplemental Figure 7E and 7F; Supplemental Figure 6C and 6D) and that subsequent invasive hyphal expansion was largely abolished (Supplemental Figure 8). Together, these findings demonstrate that m^5^C modification at C960 and C1498 in *ATG5* mRNA and at C2233 in *ATG16* mRNA is critical for appressorium maturation and full virulence in *M. oryzae*.

### Ncl1 is a downstream effector of the Pmk1–MAPK signaling cascade

Given Ncl1’s essential role in mRNA m^5^C methylation during appressorium maturation and autophagy in the rice blast fungus, we hypothesized that Ncl1 is a downstream effector of the Pmk1–MAPK signaling pathway. To test this, we first validated the *in vivo* interaction between Pmk1-GFP and Ncl1-FLAG by co-immunoprecipitation (Supplemental Figure 9A). In wild-type extracts, we observed an additional, slower-migrating band above the Ncl1-GFP signal; this band was absent in the Δ*pmk1* mutant and disappeared after alkaline phosphatase treatment, indicating that it represents phosphorylated Ncl1-GFP (Supplemental Figure 9B).

Mass spectrometric analysis identified three putative Pmk1-dependent phosphorylation sites on Ncl1: S491, T492, and T579 (Supplemental Figure 9C). Substitution of these residues with alanine (S491A/T492A and T579A) reduced the intensity of the phospho-band (Supplemental Figure 9D), confirming that Pmk1 phosphorylates Ncl1 at these positions. Steady-state levels of Ncl1 protein were markedly lower in the Δ*pmk1* mutant and the phospho-site mutants than in the wild type or complemented strains (Supplemental Figure 9E and 9F), demonstrating that Pmk1-mediated phosphorylation is critical for Ncl1 stability.

Despite the reduction in Ncl1 abundance and phosphorylation, Pmk1 activity did not influence the subcellular localization of Ncl1-GFP, which remained exclusively nuclear during appressorium development in both wild-type and Δ*pmk1* backgrounds (Supplemental Figure 9G). Pathogenicity assays revealed that the phospho-deficient allele *NCL1*^*S491A/T492A/T579A*^ conferred significantly reduced virulence compared with the fully complemented strain (Supplemental Figure 10A and 10B). RNA dot blot quantification showed that global m^5^C levels in this phospho-deficient mutant were likewise substantially lower than in the complemented control (Supplemental Figure 10C and 10D), indicating that Pmk1-mediated phosphorylation of Ncl1 is required for full m^5^C methyltransferase activity, maintenance of Ncl1 protein levels, and complete virulence.

To further explore the effects of phosphorylation on Ncl1 beyond its role in protein stability, we expressed C-terminally His-tagged Ncl1^S491A/T492A/T579A^ in *Escherichia coli*, purified the protein, and used it for in *vitro* methyltransferase activity assays with wild-type Ncl1 as a control. The methylation reaction system was established following the protocol of the MTase-Glo Methyltransferase Assay Kit (Promega, catalog # V7601), with a 26-nt RNA oligonucleotide containing the validated *ATG5* m^5^C site (see Figure 6A) as the substrate. After 60 min of incubation at 23°C, the reaction products were spotted onto an Amersham Hybond-N+ membrane and probed with an anti-m^5^C antibody (A22062, Abclonal, 1:1000). Quantification of signal intensity using ImageJ revealed that Ncl1^S491A/T492A/T579A^ exhibited significantly reduced methyltransferase activity compared with wild-type Ncl1 (Supplemental Figure 12D). We further analyzed the expression and subcellular localization of Ncl1^S491A/T492A/T579A^-GFP during appressorium formation. Confocal laser scanning microscopy showed that its subcellular localization was unchanged relative to that of wild-type Ncl1-GFP (Supplemental Figure 12E), whereas western blot analysis revealed that its abundance was significantly reduced in appressoria (Supplemental Figure 12F). These results demonstrate that Pmk1-mediated phosphorylation fine-tunes Ncl1 function by regulating both its catalytic activity and protein abundance without affecting its subcellular localization.

In summary, our findings confirm Ncl1 as a downstream target of the Pmk1–MAPK pathway; Pmk1-mediated phosphorylation modulates Ncl1 stability and catalytic activity without affecting its nuclear localization, supporting a role for Pmk1 in the intricate regulation of appressorium maturation in *M. oryzae*.

### Ncl1 stability is controlled by SUMOylation

*In silico* scanning using the GPS-SUMO server revealed 12 high-scoring SUMOylation sites and two SUMO-interaction motifs—designated SIM1 (VVADV) and SIM2 (LLQLP)—within Ncl1 (Supplemental Figure 9H). Based on these predictions, and given that mammalian NSUN2 is stabilized and retained in the nucleus through SUMO-2/3 modification (Hu et al., 2021), we hypothesized that a similar post-translational mechanism controls Ncl1 stability in *M. oryzae*. Co-immunoprecipitation using the wild-type strain P131 co-expressing Smt3-FLAG and Ncl1-GFP confirmed an *in vivo* interaction between Smt3 and Ncl1 (Supplemental Figure 9I).

To test SUMOylation directly, we performed an *in vitro* reaction containing purified GST-Ncl1, SUMO E1 (Aos1–Uba2-MBP), SUMO E2 (Ubc9-MBP), His-Smt3, ATP, and MgCl_2_. After 2 h at 37°C, the products were purified and analyzed by western blot using anti-His and anti-GST antibodies. High-molecular-weight, poly-SUMOylated GST-Ncl1 was detected only in the presence of His-Smt3; no such signal was observed in the negative control lacking His-Smt3 (Supplemental Figure 9J). To investigate Ncl1 SUMOylation *in vivo*, we expressed Ncl1-GFP in wild-type and Δ*smt3* backgrounds. Immunoprecipitation with anti-GFP beads followed by western blotting revealed abundant poly-SUMOylated Ncl1 in the WT, whereas this signal was markedly reduced in the Δ*smt3* mutant (Supplemental Figure 9K), confirming that Smt3-mediated SUMOylation of Ncl1 occurs in *M. oryzae*.

Assessment of protein stability showed that steady-state levels of Ncl1 were markedly lower in the Δ*smt3* mutant than in the WT (Supplemental Figure 9L and 9M), indicating that SUMOylation positively regulates Ncl1 abundance. Despite this difference, subcellular localization was unaffected: Ncl1-GFP remained exclusively nuclear during appressorium development in both wild-type and Δ*smt3* strains (Supplemental Figure 9N).

We next constructed the *NCL1*^*ΔSIM1ΔSIM2*^ mutant, in which both SUMO-interaction motifs are deleted. Virulence assays and RNA dot blot analysis revealed that, relative to the fully complemented strain, the SIM deletion mutant exhibited a significant decrease in global m^5^C abundance and a pronounced reduction in pathogenicity (Supplemental Figure 10A–10D). Therefore, phosphorylation and SUMOylation act in concert to fine-tune Ncl1 function: loss of either modification—by blocking phosphorylation at S491/T492/T579 or eliminating SIM-dependent SUMOylation—reduces RNA m^5^C levels, destabilizes Ncl1, and attenuates virulence. These findings reveal a regulatory nexus in which two distinct post-translational modifications act on an RNA-modifying enzyme to control fungal pathogenicity.

To confirm that the observed phenotypes depend on the methyltransferase activity of Ncl1, we generated the catalytically inactive allele *NCL1*^*C353A*^ and expressed it under the native *NCL1* promoter in the Δ*ncl1* background. The resulting strain, Δ*ncl1/NCL1*^*C353A*^, failed to restore (1) wild-type pathogenicity on rice and barley (Supplemental Figure 10A and 10B), (2) global m^5^C levels as measured by RNA dot blot (Supplemental Figure 10C and 10D), (3) normal appressorial turgor (Supplemental Figure 10E and 10F), or (4) Atg8–PE accumulation (Supplemental Figure 10G). In contrast, the complemented Δ*ncl1/NCL1* strain showed full rescue of all defects. Consistent with this, *in vitro* methyltransferase assays showed significantly reduced methyltransferase activity in Ncl1^C353A^ compared with wild-type Ncl1 (Supplemental Figure 12D), confirming that residue C353 is essential for Ncl1’s full catalytic activity. These data demonstrate that the biological effects described above depend on Ncl1-mediated m^5^C catalysis.

## Discussion

**Figure 7:**
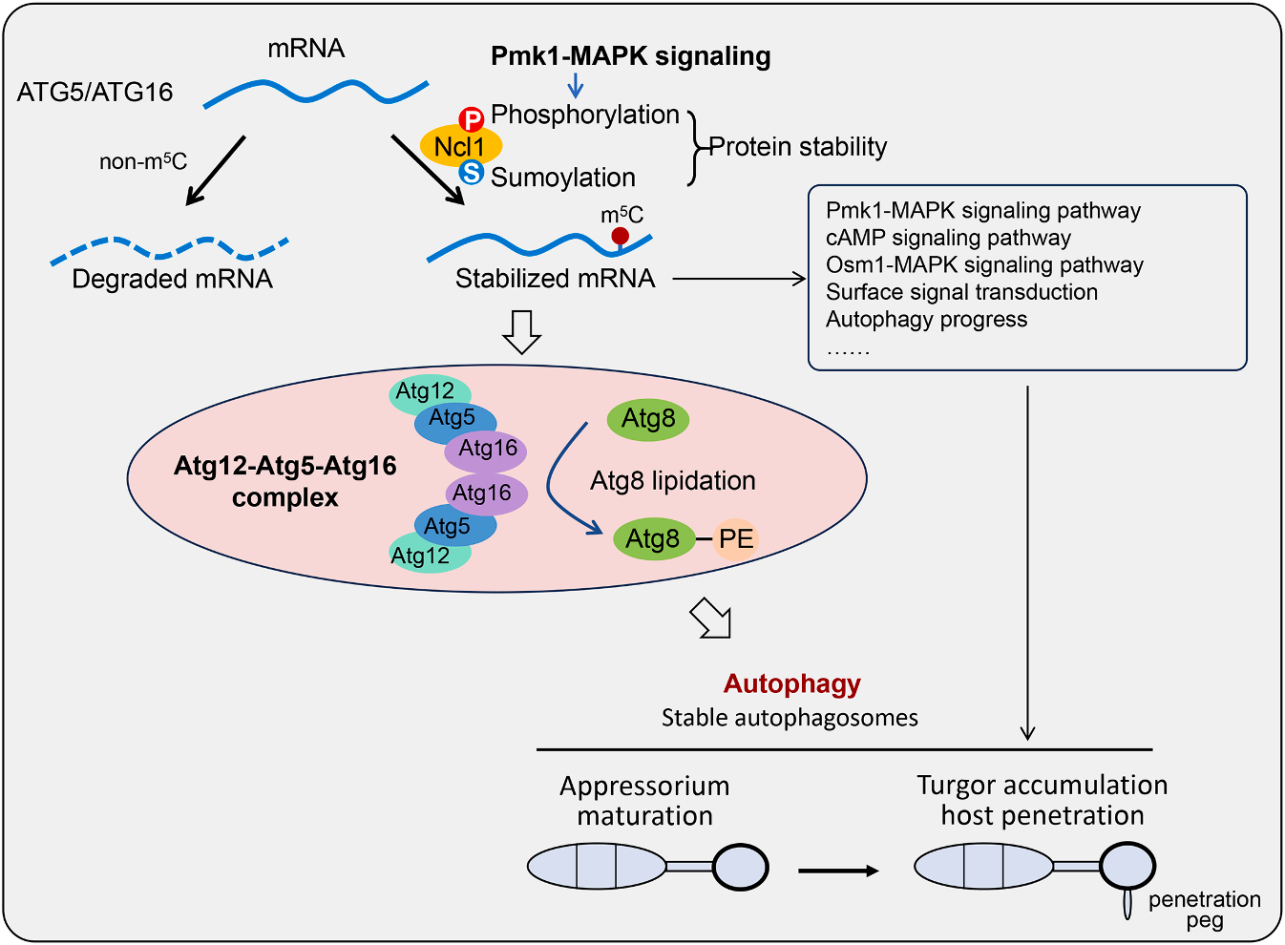
Proposed regulatory mechanism of Ncl1-mediated m^5^C methylation during *M. oryzae* infection. As a global regulatory factor, m^5^C methylation exerts multi-layered control over *M. oryzae* infection. It directly stabilizes the mRNAs of *ATG5* and *ATG16*, ensuring that protein levels are sufficient to form the Atg12–Atg5–Atg16 complex, which is essential for promoting Atg8 lipidation. The lipidation of Atg8 facilitates its association with autophagosomes and maintains autophagosomal stability, thereby ensuring efficient autophagy. Autophagy, in turn, promotes the maturation of *M. oryzae* appressoria, enabling them to accumulate sufficient turgor pressure to drive the formation of infection pegs that penetrate the host epidermis and achieve successful infection. In parallel, m^5^C broadly regulates key signaling and physiological pathways, including the Pmk1–MAPK, cAMP, and Osm1–MAPK signaling pathways, surface signal transduction, and autophagy progression, collectively fine-tuning appressorium maturation and host penetration capacity. In addition, the stability of the m^5^C methyltransferase Ncl1 is co-regulated by Smt3-mediated SUMOylation and Pmk1 pathway-mediated phosphorylation, ensuring proper Ncl1 function in m^5^C methylation.

Functional studies of m^6^A have established the broad regulatory importance of mRNA modification in eukaryotes, and recent work in *M. oryzae* has reinforced this view (Ren et al., 2022). In contrast, another widespread post-transcriptional mRNA modification, m^5^C, remains poorly characterized, especially in plant-pathogenic fungi. Here, we identify the m^5^C RNA methyltransferase Ncl1 as a pivotal regulator of virulence and appressorium development in *M. oryzae*. We demonstrate that m^5^C modification enhances the stability of *ATG5* and *ATG16* mRNAs, thereby ensuring adequate protein levels for assembly of the Atg12–Atg5–Atg16 complex that drives Atg8 lipidation. This lipidation is essential for Atg8 association with autophagosomal membranes and thus for productive autophagy. Autophagy, in turn, underpins appressorium maturation, which enables the fungus to generate the turgor pressure required for infection peg formation and host penetration, ultimately leading to successful infection. We further show that Ncl1 protein stability is governed by Pmk1-mediated phosphorylation and Smt3-dependent SUMOylation (Figure 7). Our findings illuminate the interplay between epigenetic marks and post-translational control in fungi, revealing a central role for m^5^C RNA methylation in the regulation of autophagy and pathogenicity in *M. oryzae*.

Our study highlights the pivotal role of RNA modification in fungal pathogenicity, particularly during appressorium formation in *M. oryzae*. We found that Ncl1-mediated m^5^C methylation stabilizes the mRNAs of autophagy-related genes (e.g., *ATG5* and *ATG16*), a process essential for intracellular recycling and efficient host infection. In contrast, accumulating evidence indicates that m^6^A modification exerts the opposite effect, accelerating the decay of *ATG* transcripts and thereby restraining autophagy. METTL3-mediated m^6^A methylation negatively regulates autophagy to maintain porcine blastocyst development, and knockdown of METTL3 enhances *ATG5* mRNA stability (Cao et al., 2021). Likewise, the m^6^A demethylase FTO targets *ULK1* (*ATG1*) transcripts and increases ULK1 protein abundance (Jin et al., 2018), whereas in mice, FTO protects *ATG5* and *ATG7* mRNAs from degradation by removing m^6^A modifications (Wang et al., 2020). In *M. oryzae*, the m^6^A writer Mta1 promotes degradation of *ATG8* mRNA, thereby limiting autophagy activity (Ren et al., 2022). Our supplementary RT–qPCR assays confirmed that *ATG8* is significantly upregulated (by approximately 2.6-fold) in the Δ*mta1* mutant during appressorium formation, whereas *ATG5* and *ATG16* expression is unchanged (Supplemental Figure 12A), further supporting gene-specific regulation of autophagy-related genes by m^6A^. Together, Ncl1-mediated m^5^C and Mta1-mediated m^6^A modifications exhibit gene-specific, functionally antagonistic, regulation of autophagy during *M. oryzae* appressorium formation, representing a precise dual-checkpoint system for tuning autophagy activity to the optimal level for host infection. This antagonism arises from their opposing effects on autophagy—m^5^C promotes the initiation of basal autophagy, whereas m^6^A restricts excessive autophagy—rather than direct reciprocal regulation of the same target genes. Functionally, m^5^C acts as a positive regulator that directly stabilizes *ATG5* and *ATG16* mRNAs via two novel, functionally validated cytosine sites (C960/C1498 in *ATG5* and C2233 in the *ATG16* 3′ UTR), which have not been previously reported in any organism. This m^5^C-mediated stabilization ensures sufficient levels of *ATG5* and *ATG16* expression to drive Atg8 lipidation, autophagosome assembly, and the initiation of basal autophagy, and genetic replacement of these sites phenocopies the virulence defects of Δ*atg5* and Δ*atg16* mutants, demonstrating that single cytosine methylation events can control autophagy-dependent infection. In contrast, m^6^A is a negative regulator that targets the autophagy marker gene *ATG8*. The two modifications therefore act on complementary components of the autophagy machinery. Notably, both regulate autophagy via the conserved mechanism of mRNA stability modulation, but with opposite effects: Ncl1-mediated m^5^C methylation slows the degradation of *ATG5* and *ATG16* transcripts (actinomycin D assay; Supplemental Figure 4A and 4B), a stabilizing mode unique to m^5^C in fungi, whereas m^6^A typically destabilizes its target *ATG* transcripts.

Our functional validation of m^5^C sites in non-ATG genes related to pathogenicity further demonstrates the global regulatory significance of Ncl1-mediated m^5^C methylation in *M. oryzae* infection. By integrating high-confidence m^5^C MeRIP-seq peaks with *in silico* predictions (http://www.rnanut.net/rnam5Cfinder/), we identified m^5^C sites in key regulators of two central pathogenicity pathways: the Pmk1–MAPK pathway (PMK1, C1990) and the cAMP pathway (CPKA, C3013/C3244) (Supplemental Figure 11A). To investigate the functional roles of these m^5^C marks, we introduced silent C→T substitutions into the respective deletion mutants at the identified sites, creating Δ*pmk1/PMK1*^*C1990T*^ and Δ*cpka/CPKA*^*C3013T/C3244T*^. Although complementation with alleles carrying m^5^C site mutations restored appressorium formation capacity to levels similar to the WT, these strains exhibited significant impairments in appressorium maturation and insufficient turgor accumulation—indicated by increased collapse rates following osmotic stress (15% glycerol treatment; Supplemental Figure 11C and 11D)—and reduced virulence relative to both the wild-type strain and the corresponding full-complementation strains (Supplemental Figure 11G). Collectively, these findings establish a direct causal relationship between m^5^C methylation of *PMK1* and *CPKA* transcripts and the full pathogenicity of *M. oryzae*, whereby m^5^C modification modulates appressorium maturation and turgor generation as downstream regulatory outputs of the Pmk1–MAPK and cAMP signaling cascades. To further investigate the scope of m^5^C-dependent regulation, we validated m^5^C sites in two additional non-ATG virulence factors with well-characterized roles in *M. oryzae* pathogenicity: SMO1, a Ras GTPase-activating protein critical for Ras signal termination (Kershaw et al., 2019), and LDP1, a lipid droplet-associated protein required for full pathogenicity (Cai et al., 2020). We identified one high-confidence m^5^C site in SMO1 (C3928) and two in LDP1 (C321/C323) (Supplemental Figure 11B); silent mutations at these sites similarly resulted in significant virulence attenuation relative to the wild-type and full-complementation strains (Supplemental Figure 11H). Overall, these data indicate that Ncl1-mediated m^5^C methylation regulates at least four distinct virulence modules (MAPK signaling, cAMP signaling, Ras signal termination, and lipid droplet metabolism) and extends beyond autophagy to coordinate multiple key cellular processes essential for appressorium maturation and successful host infection. This broad regulatory spectrum indicates that m^5^C methylation is not restricted to autophagy genes but functions as a global post-transcriptional mechanism that regulates the expression of diverse pathogenicity determinants in *M. oryzae*.

In *M. oryzae,* Ncl1-directed m^5^C methylation is required to preserve the stability of ATG5 and ATG16 mRNAs, both of which are essential for autophagy. The Atg12–Atg5–Atg16 complex governs Atg8 lipidation, a reaction that maintains Atg8–PE levels and thereby drives autophagy initiation (Suzuki et al., 2001; Fujita et al., 2008). Our data demonstrate that site-specific m^5^C methylation is indispensable for proper autophagy, as mutants lacking these cytosine marks exhibit reduced Atg8–PE levels and fewer autophagosomes. Interestingly, *M. oryzae* exhibits behavior distinct from that of yeast and mammals: Atg8 still forms puncta in the absence of *ATG5* and *ATG16*, implying a potential contribution of the Cvt pathway to Atg8 recruitment (Baba et al., 1994, 1997; Suzuki et al., 2007). Because Atg5 membrane binding is essential for both autophagy and the Cvt pathway (Walczak and Martens, 2013), its impairment in these mutants underscores the critical role of m^5^C in maintaining *ATG* mRNA stability and facilitating autophagy in *M. oryzae*. Overall, our findings identify a novel mechanism regulating fungal autophagy, with direct implications for the pathogen’s survival and infection strategy.

The RNA methyltransferase Ncl1 in *M. oryzae* is governed by a dual regulatory mechanism involving phosphorylation and SUMOylation, both of which are essential for its stability and function. Phosphorylation by the Pmk1 signaling pathway, a central regulatory pathway in eukaryotic cells (Wilson and Talbot, 2009; Osés-Ruiz et al., 2021), is required to maintain Ncl1 stability and modulate the pathogen’s epigenetic responses during infection. SUMOylation, the covalent attachment of SUMO proteins to target lysines, likewise preserves Ncl1 stability, a prerequisite for its methylation of target transcripts. These findings deepen our understanding of fungal epigenetics and underscore the importance of post-translational modifications in pathogenicity. The coordinated action of phosphorylation and SUMOylation ensures that Ncl1 functions precisely when and where it is needed, supporting the dynamic, context-specific processes that underpin *M. oryzae* infection and development. Notably, the core Pmk1–MAPK cascade phosphorylates Ncl1, thereby determining its protein stability. This finding reveals that, in addition to regulating transcription through factors such as Mst12 and Hox7 (Osés-Ruiz et al., 2021), the Pmk1–MAPK pathway also influences mRNA abundance through the RNA modification factor Ncl1. Consequently, the Pmk1–MAPK–Ncl1 regulatory module constitutes a novel epigenetic control circuit that orchestrates the formation of fungal infection structures and, by extension, the entire pathogenic process.

Notably, m^5^C RNA methylation plays a previously unrecognized role in mediating fungal–plant interactions by enabling *M. oryzae* to adapt to the dynamic host microenvironment. During infection, plants activate defense responses such as reactive oxygen species bursts and nutrient sequestration, which disrupt fungal cellular homeostasis. Our data show that Ncl1-mediated m^5^C stabilizes mRNAs of core virulence genes (e.g., *ATG5*, *ATG16*, *PMK1*, and *CPKA*), acting as a “post-transcriptional buffer” to ensure robust expression of these genes under host-derived stress. This regulatory strategy allows *M. oryzae* to maintain critical processes (such as autophagy, appressorium turgor accumulation, and signal transduction) even when transcriptional programs are perturbed, thereby facilitating successful host colonization. Ncl1-mediated m^5^C methylation represents a promising target for novel antifungal strategies to control rice blast disease. Conventional fungicides often target metabolic enzymes or cell wall components, leading to rapid evolution of resistance through single-gene mutations. In contrast, targeting Ncl1 or its downstream m^5^C modification sites offers pleiotropic effects: disrupting this epigenetic pathway compromises multiple virulence modules (namely autophagy, MAPK/cAMP signaling, and lipid metabolism) simultaneously, thereby reducing the likelihood of resistance. Feasible translational approaches include (1) designing small-molecule inhibitors that target Ncl1’s SAM-binding domain or catalytic core (C353 residue) to block its methyltransferase activity and (2) developing host-induced gene silencing constructs to suppress Ncl1 expression or disrupt key m^5^C sites in virulence-related mRNAs. These strategies are environmentally sustainable and could be extended to other plant-pathogenic fungi harboring Ncl1 homologs, providing a broad-spectrum solution for fungal disease control.

In conclusion, our study clarifies the essential function of Ncl1-catalyzed m^5^C RNA methylation in governing autophagy and pathogenicity in *M*. *oryzae*, thereby uncovering a previously unknown epigenetic regulatory circuit that links post-transcriptional modification to fungal infection. These findings deepen our understanding of fungal biology and identify prospective molecular targets for the development of antifungal interventions to manage diseases caused by plant-pathogenic fungi.

## Methods

### Fungal strains and growth conditions

The *M*. *oryzae* wild-type strain P131 served as the reference strain. Details of all strains and the vectors used for genetic modifications are provided in Supplemental Tables 4 and 5. Mycelial growth of P131 and its derivative strains was carried out on OTA plates at 28°C. For protein and nucleoside extraction, vegetative mycelia were harvested from liquid CM.

### Gene knockout and complementation

The wild-type strain P131 served as the background for all genetic manipulations. The split-PCR approach was used to generate knockouts of *NCL1* and the *ATG* genes, all of which are pivotal for fungal pathogenicity; this method enabled the precise deletion of targeted loci within the *M. oryzae* genome. To confirm the functions of *NCL1* and the *ATG* genes, a complementation strategy was used in which each knockout strain was transformed with a construct carrying the corresponding wild-type allele, thereby reintroducing a fully functional copy of the gene into the mutant background.

### m^5^C-seq analysis

m^5^C-IP-seq, also referred to as MeRIP-seq, was performed by CloudSeq (Shanghai, China). Total RNA was isolated and fragmented into approximately 200-nt-long fragments using RNA Fragmentation Reagents. For immunoprecipitation, the m^5^C-IP kit (GenSeq) was used: protein A/G beads were conjugated to m^5^C-specific antibodies and incubated for 60 min at room temperature. The fragmented RNA was then incubated with the antibody-coated beads at 4°C for 4 h to promote RNA–antibody complex formation. Following incubation, the complexes were treated with proteinase K to digest proteins, and RNA was recovered using phenol:chloroform extraction. The library was prepared using the NEBNext Ultra II Directional RNA Library Prep Kit (New England Biolabs) according to the manufacturer’s instructions. Library quality was assessed using an Agilent 2100 Bioanalyzer, and sequencing was performed on an Illumina NovaSeq platform, enabling genome-wide identification and quantification of m^5^C methylation sites.

### RNA-seq analysis

RNA-seq was performed by CloudSeq Biotech (Shanghai, China). Total RNA was extracted, and ribosomal RNA was depleted using the NEBNext rRNA Depletion Kit (New England Biolabs, MA, USA) to enrich for mRNA. Strand-specific RNA libraries were subsequently prepared using the NEBNext Ultra II Directional RNA Library Prep Kit. Library quality was assessed using the Bioanalyzer 2100 system, and sequencing was performed on an Illumina NovaSeq platform, generating 150-bp paired-end reads.

### Dot blot assay

The dot blot assay was performed using total RNA isolated from WT and Δ*ncl1* mutant strains. RNA samples were added to a Hybond-N+ membrane and dried at 37°C for 30 min. The membrane was then cross-linked for 5 min using an HL-2000 HybriLinker to immobilize the RNA. After cross-linking, the membrane was blocked with 5% milk in phosphate-buffered saline with Tween-20 (PBST) for 1 h, followed by incubation with the primary anti-m^5^C antibody (A22062, Abclonal) to detect m^5^C modification. The membrane was washed with PBST to remove unbound primary antibody and incubated with the secondary antibody for 1 h at room temperature. Signals were then developed using an enhanced chemiluminescence detection system (Amersham Biosciences, Piscataway, NJ, USA). The resulting blot was quantified using ImageJ to determine m^5^C levels.

### Real-time RT–qPCR

RT–qPCR was used to determine the expression levels of autophagy-related (*ATG*) genes across *M. oryzae* developmental stages. To this end, samples representing different fungal growth phases were prepared: vegetative hyphae grown in CM for 36 h, appressoria following induction on hydrophobic membranes for 6 h, and vegetative hyphae initially grown in CM for 36 h and subsequently transferred to nitrogen-deficient medium (MM-N) for an additional 6 h. Total RNA was extracted from each sample and used for cDNA synthesis. RT–qPCR reactions were performed using SYBR Green PCR Master Mix (Takara Bio, Dalian, China) on an ABI 7500 real-time PCR system (Applied Biosystems, Foster City, CA, USA). This approach enabled accurate quantification of transcript abundance and provided insight into the regulatory dynamics of *ATG* genes throughout the developmental cycle of *M. oryzae*.

### MeRIP–qPCR quantification of m^5^C levels

Total RNA (>100 μg) or purified mRNA (>3 μg) was fragmented to approximately 200 nt by incubation at 70°C for 5 min in 10× fragmentation buffer, followed by the addition of stop buffer and ethanol precipitation of RNA. The fragmented RNA was resuspended in 50 μl of nuclease-free water and then divided into two equal parts; one part served as the input control, and the other was subjected to methylated RNA immunoprecipitation (MeRIP) using the GenSeq m^5^C MeRIP Kit (GenSeq, Shanghai, China). For MeRIP, 1–3 μg of fragmented RNA was combined with 25 μl of Protein G Magnetic (PGM) beads pre-conjugated to 2 μl of m^5^C antibody or control immunoglobulin G in 200 μl of 1× IP buffer and incubated at 4°C for 1 h. After washing, RNA was eluted with Dig solution at 55°C for 45 min, further purified using MS beads, and eluted in 10 μl of nuclease-free water. Input and immunoprecipitated samples were used immediately for RT–qPCR analysis or stored at −80°C.

### Observation of autophagy and western blot

For autophagy observation and subsequent western blot analysis, spores of each strain were inoculated into CM liquid medium and incubated at 28°C with shaking at 160 rpm for 36 h to allow germination and hyphal growth. The mycelia were then transferred to nitrogen-deficient MM-N medium and incubated for 2 or 5 h to induce autophagy. The localization and abundance of GFP–Atg8, a marker of autophagic bodies, were examined by fluorescence microscopy as indicators of autophagy induction. In parallel, spore suspensions were directly inoculated onto hydrophobic slides to assess the initial state of autophagy; the distribution and intensity of GFP–Atg8 fluorescence were recorded at 0, 2, and 4 hpi to track autophagy dynamics.

For western blot analysis, replicate cultures were grown in CM liquid medium under the same conditions and shifted to MM-N medium for 0, 2, or 5 h to synchronize autophagy progression, after which total protein extracts were prepared from the mycelia. Equal amounts of protein were separated by SDS–PAGE according to molecular weight, and western blotting was performed using a primary anti-GFP antibody (1:5000 dilution, Sigma, USA) to detect GFP–Atg8. The blot was developed to visualize the GFP signal, which was quantified to assess autophagy levels under different nitrogen conditions.

### Quantitative assessment of autophagic flux using E64d and pepstatin A

For quantitative measurement of autophagic flux, conidial suspensions of WT, Δ*ncl1*, Δ*atg5*, Δ*atg16*, Δatg5/*ATG5*^C960T/C1498T^, and Δatg16/*ATG*16^C2233T^ strains, all expressing GFP–Atg8, were inoculated into CM liquid medium and cultured at 28°C with shaking at 160 rpm for 36 h. Mycelia were harvested, washed twice with MM-N medium, and divided into two equal groups: the inhibitor group was transferred to MM-N medium containing 10 μg/ml E64d and 10 μg/ml pepstatin A, and the mock group was transferred to MM-N medium containing an equal volume of DMSO. Both groups were incubated at 28°C with shaking at 160 rpm for 3 h. Total protein was extracted using radioimmunoprecipitation assay (RIPA) lysis buffer supplemented with 1 mM PMSF and 1× protease inhibitor cocktail. Equal amounts of protein were separated using 12% SDS–PAGE, transferred to a polyvinylidene fluoride membrane, and immunoblotted with anti-GFP and anti-GAPDH antibodies. Signal intensities of full-length GFP-Atg8 and free GFP were quantified using ImageJ. Autophagic flux was calculated as follows: autophagic flux = (free GFP intensity in the inhibitor group – free GFP intensity in the mock group)/total GFP intensity (GFP-Atg8 + free GFP) in the mock group.

### Pathogenicity tests and infection assay

To assess the pathogenicity of each *M. oryzae* strain, spray-infection assays were conducted on barley and rice. For rice, a conidial suspension was adjusted to 5 × 10^4^ conidia ml^−1^ with double-distilled H_2_O, and 5 ml of the suspension was uniformly sprayed onto 2-week-old seedlings (*Oryza sativa* cv. LTH). The inoculated seedlings were incubated at 28°C and 90% relative humidity, with darkness for the first 24 h followed by a 12 h light/12 h dark cycle. Disease lesions were monitored and photographed daily. For barley, 2 ml of a conidial suspension (3 × 10^4^ conidia ml^−1^) was sprayed onto 7-day-old leaves (*Hordeum vulgare* cv. E9), and infected leaves were photographed 5 days post inoculation. Each pathogenicity test was performed independently three times, and leaves were harvested to measure blast lesion size. To monitor the infection process microscopically, a conidial suspension (1 × 10^5^ spores ml^−1^ in double-distilled H_2_O) was injected into rice leaf sheaths. The inoculated samples were maintained at 28°C and 90% humidity, and infection progression was recorded at successive time points. Penetration and invasive hyphal growth were visualized using a Nikon Ni90 microscope to provide detailed information on early infection events.

### Appressorium formation and collapse assays

To evaluate appressorium formation, conidial suspensions were prepared at a density of 5 × 10^4^ spores ml^−1^ and evenly spread onto a hydrophobic surface. Samples were incubated in the dark for 24 h under conditions favorable for *M. oryzae* development. After incubation, at least 300 appressoria per strain were microscopically examined to assess the efficiency of appressorium formation. In addition, the structural stability of mature appressoria was tested by exposing them to an 8% glycerol solution for 30 min; this treatment was applied to appressoria that had fully matured on the hydrophobic surface. Following glycerol exposure, the samples were examined to determine the percentage of collapsed appressoria, providing a measure of their structural integrity and potential functionality during infection.

### Phos-tag assay

The Ncl1-GFP fusion protein was introduced into both the P131 strain and the Δ*pmk1* mutant, whereas the phospho-deficient alleles Ncl1^S491A/T492A^-GFP and Ncl1^T579A^-GFP were introduced into the Δ*ncl1* background. For protein analysis, mycelial samples were subjected to western blotting with anti-GFP and anti-GAPDH antibodies. Proteins were resolved on 8% SDS–polyacrylamide gels containing 5.0 mM Phos-tag AAL and 10 mM MnCl_2_ according to the procedure of Shindo et al. (2016). Electrophoresis was performed at 20 mA for 4–5 h. Gels were then washed three times for 10 min each in transfer buffer supplemented with 10 mM EDTA, followed by a single 10-min wash without EDTA. Proteins were transferred from the Mn^2+^-Phos-tag gel to a polyvinylidene fluoride membrane at 45 V overnight at 4°C. The membrane was subsequently probed with an anti-GFP antibody to detect phosphorylation-dependent mobility shifts.

### Co-immunoprecipitation assay

The pKNRG-Pmk1 and pKNRG-Ncl1 constructs, both driven by their native promoters, were co-transformed into the wild-type strain with pKNFLAG-Ncl1 and pKNFLAG-Smt3, respectively. Verified transformants were cultured in liquid CM at 28°C with shaking at 150 rpm for 48 h. Approximately 1 g of fresh mycelia was harvested and rinsed with sterile water, and total protein was extracted using IP cell lysis buffer (Biyuntian, Beijing, China). The lysate was incubated at room temperature for 2 h with 10 μl of anti-FLAG magnetic beads (Bimake, Beijing, China) to capture FLAG–Pmk1 or FLAG–Ncl1 protein complexes. The beads were then washed three times with PBST to remove unbound proteins, followed by three washes with 50 mM trimethylammonium bicarbonate (pH 8.5). Immunoprecipitated proteins were eluted with 100 μl of elution buffer, separated by SDS–PAGE, and analyzed by western blot using appropriate antibodies to detect interacting proteins.

### *In vitro and in vivo* poly-SUMOylation assays

To determine whether Ncl1 is SUMOylated by Smt3 *in vitro*, a reaction mixture was prepared containing purified GST-Ncl1, SUMO E1 (Aos1–Uba2-MBP), SUMO E2 (Ubc9-MBP), His-Smt3, and reaction buffer supplemented with ATP and MgCl_2_. After incubation for 2 h at 37°C, the products were purified and analyzed by western blot using anti-His and anti-GST antibodies. To assess *in vivo* SUMOylation, the pKNRG-Ncl1 construct was introduced into both wild-type and Δ*smt3* strains. Total protein extracts from WT/*NCL1*-GFP and Δ*smt3*/*NCL1*-GFP mycelia were incubated with anti-GFP nanobody agarose beads (KTSM1301) to capture Ncl1-GFP. SUMOylation was detected by western blot using an anti-Smt3 polyclonal antibody (Biorbyt, orb351113), with an anti-GFP antibody (Biodragon, China) as the loading control.

### RNA m^5^C methylation assay in *vitro*

To assess the catalytic activity of Ncl1 variants, full-length coding sequences of wild-type Ncl1, the phospho-deficient mutant Ncl1^S491A/T492A/T579A^, the SUMO-interacting motif (SIM) deletion mutant Ncl1^ΔSIM1ΔSIM2^ (lacking amino acids 51–55 and 825–829), and the catalytically defective mutant Ncl1^C353A^ were cloned into the prokaryotic expression vector pET-28a with a C-terminal His-tag. Recombinant plasmids were transformed into *E. coli* BL21 (DE3) competent cells. Protein expression was induced with 0.5 mM isopropyl β-D-1-thiogalactopyranoside at 16°C for 16 h. His-tagged proteins were purified using Ni-NTA agarose (Qiagen) according to the manufacturer’s protocol, and protein purity was verified by 12% SDS–PAGE followed by Coomassie brilliant blue staining.

The *in vitro* methylation assay was performed using the MTase-Glo Methyltransferase Assay Kit (Promega, catalog # V7601) with modifications. A 26-nt RNA oligonucleotide containing the validated m^5^C site (C960) from *ATG5* (sequence: 5′-GGGUCAAGGAGGCCGACUUUGUGCCC-3′) was synthesized by Tsingke Biotechnology (Beijing, China) and used as the substrate. The reaction mixture contained 20 mM Tris–HCl (pH 8.0), 50 mM NaCl, 1 mM EDTA, 3 mM MgCl_2_, 0.1 mg/ml BSA, 1 mM dithiothreitol, 40 U ml^−1^ RNase inhibitor, 1 mM S-adenosylmethionine, 2 μg of purified Ncl1 variant protein, and 1 μg of RNA substrate. Reactions were incubated at 23°C for 60 min and terminated by heating at 95°C for 5 min.

### Statistical analysis

Statistical analyses were conducted using Excel (Microsoft, 2021) or Prism 9 (GraphPad). The exact numbers of biological replicates and the specific statistical tests used are indicated in the figure legends; each experiment was performed at least three times. Statistical significance was determined using two-sided unpaired Student’s *t-test*s or ANOVA, as appropriate. All error bars represent the standard deviation (SD) of the mean. Asterisks denote levels of statistical significance: ∗*p* < 0.05; ∗∗*p* < 0.01; ∗∗∗*p* < 0.001; ns, not significant.

## Data and code availability

• All data required to assess the findings presented in the manuscript are included within the paper itself and/or the supplemental information.
• The raw data from the m^5^C-seq and RNA-seq analyses have been deposited in the Sequence Read Archive database under the collective accession number PRJNA1185837. These data can be accessed at: https://www.ncbi.nlm.nih.gov/sra/PRJNA1185837.

## Funding

This work was supported by the 10.13039/501100012166National Key Research and Development Program of China (2023YFD1400200), the 10.13039/501100021171Guangdong Basic and Applied Basic Research Foundation (2024A1515010774), and the 10.13039/501100001809National Natural Science Foundation of China (grant 32072365).

## Acknowledgments

No conflict of interest is declared.

## Author contributions

X.-L.C. conceived the study and designed the investigation. X.C. conducted most of the experiments with assistance from H.H., C.L., and Z.R. Y.L. and Y.N. participated in partial experimental supplementation and manuscript revision. H.L., L.Z., and J.H. participated in the design of the investigation. X.-L.C. and X.Y. supervised the project. X.-L.C., X.C., and X.Y. drafted and revised the manuscript. All authors read and approved the final version.

## Supplemental information

Document S1. Supplemental Figures 1–12

Supplemental Table 1. Significantly reduced m^5^C peaks in mRNA of the Δ*ncl1* mutant

Supplemental Table 2. DEGs in the Δ*ncl1* mutant relative to the wild type

Supplemental Table 3. m^5^C-seq results for transcripts of autophagy-related genes

Supplemental Table 4. Fungal strains used in this study

Supplemental Table 5. Plasmids used in this study

Document S2. Article plus supplemental information
