# Supplementary material for: Ncl1-dependent m5C RNA methylation promotes appressorial autophagy and pathogenicity in the rice blast fungus
Source: Plant Commun. 2026 Mar 11;7(8):101817. doi: 10.1016/j.xplc.2026.101817 (PMC13477019; doi:10.1016/j.xplc.2026.101817)
Supplement: Supplementary file 1 [file mmc1.pdf]

Plant Communications, Volume 7

Supplemental information

Ncl1-dependent m^{5}C RNA methylation promotes appressorial autophagy and pathogenicity in the rice blast fungus

Xuan Cai, Hong Hu, Caiyun Liu, Zhiyong Ren, Yunfeng Li, Yanfang Nie, Hao Liu, Lu Zheng, Junbin Huang, Xiaolin Yang, and Xiao-Lin Chen

Supplementary data: Cai et al. Ncl1-mediated RNA 5-methylcytosine regulates appressorial autophagy and pathogenicity in the rice blast fungus

This Supplemental data includes:

Supplemental Figure S1 to S12
Supplemental Table S1 to S5

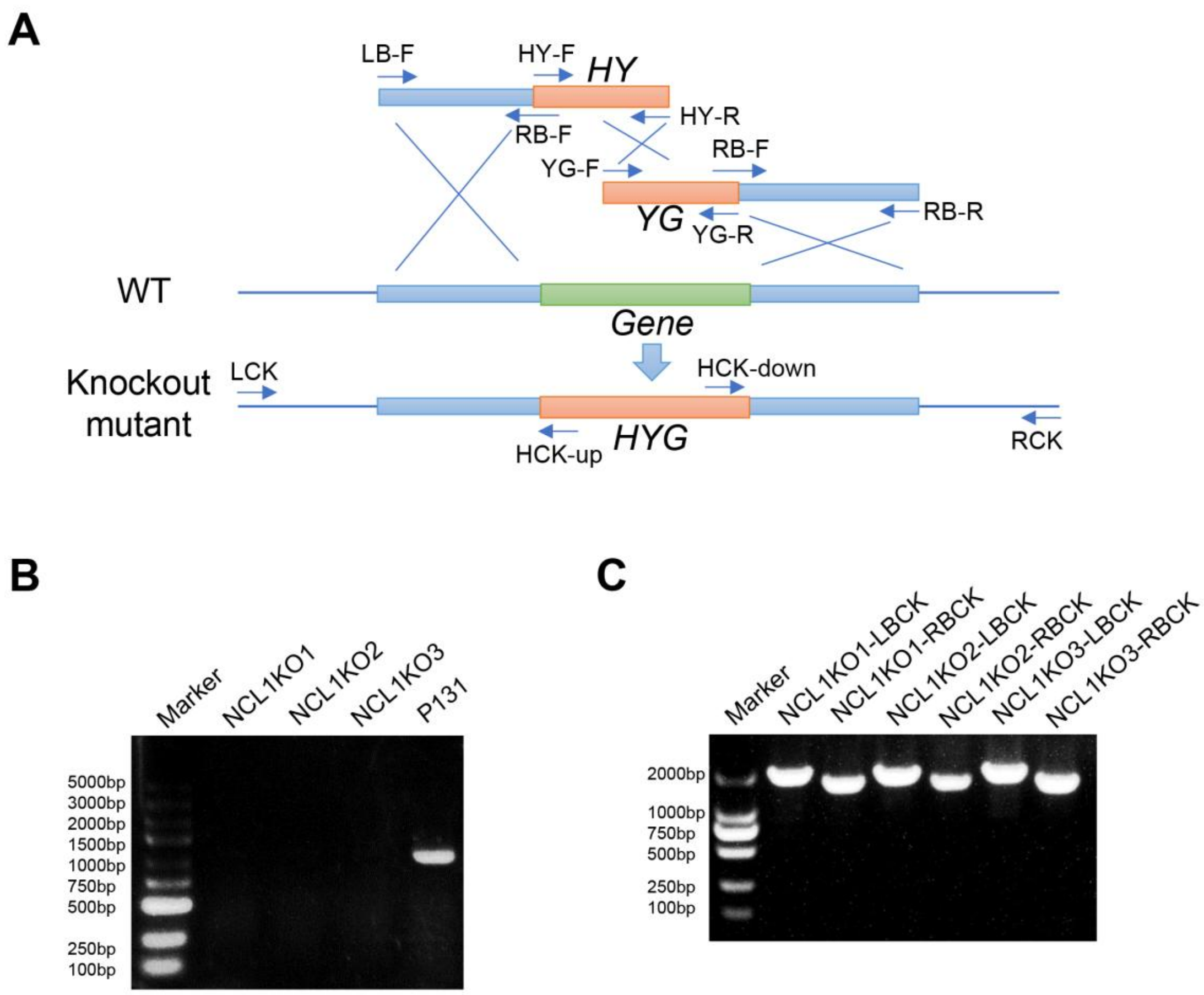

Figure S1. Gene deletion of *NCL1*. (A) The gene knockout procedure using the split-marker approach. **(B)** PCR validation was performed to amplify the expression of this gene in both the transformants and the wild-type strains. **(C)** PCR validation was conducted on the flanking sequences adjacent to the replacement fragment using the LCK/HCK-up and RCK/HCK-down primers.

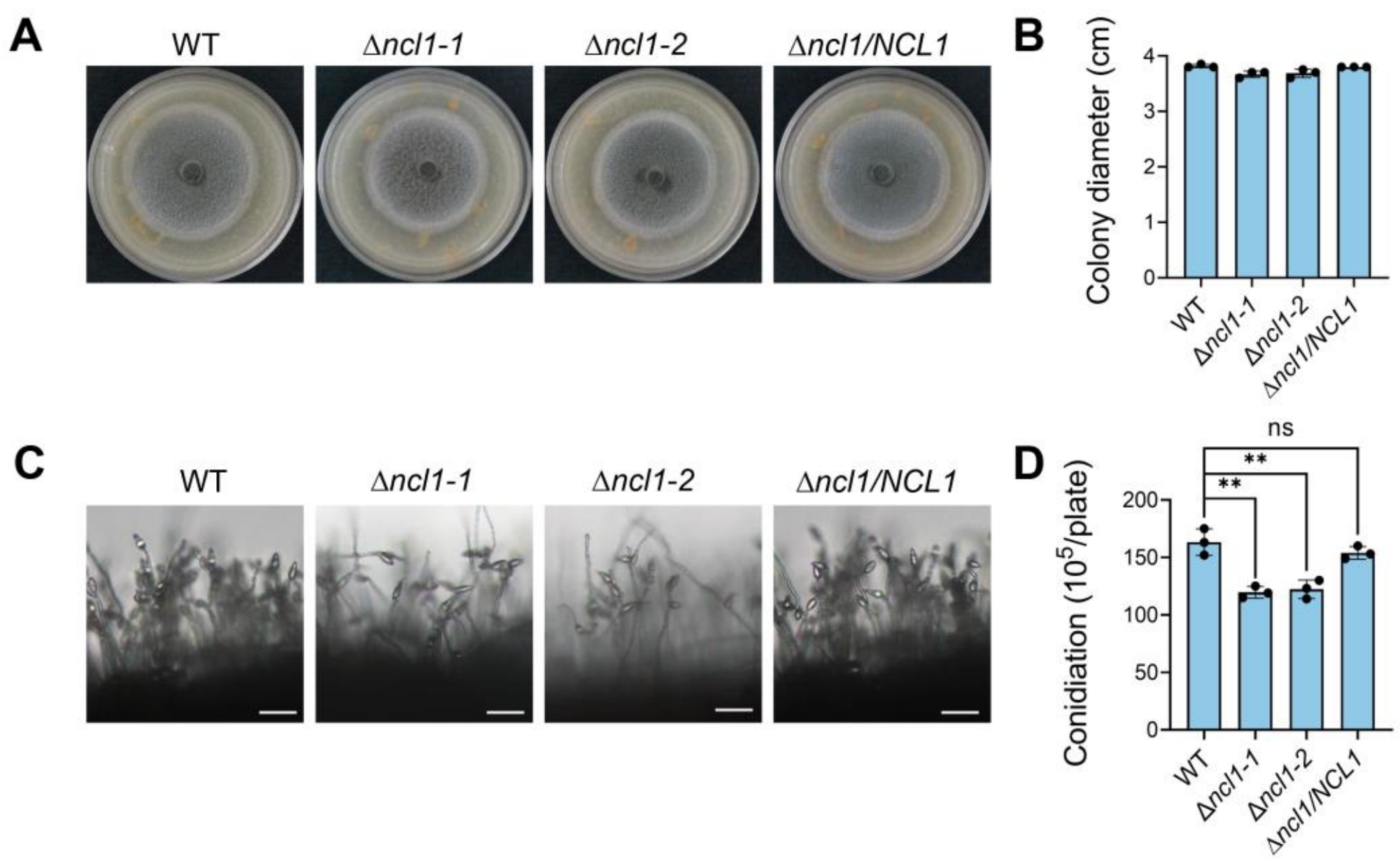

Figure S2. *NCL1* regulates conidial formation in *M. oryzae*. (A) The colony morphology of the wild-type (WT), the Δ*ncl1* mutants, and the complemented strain were observed on oatmeal tomato agar (OTA) plates at 28°C for 5 days. **(B)** The colony diameters of the different strains were measured and statistically analyzed. Data are means ± SD from 3 biological replicates (n = 3 plates per replicate). Statistical significance was determined by one-way ANOVA followed by Tukey’s HSD test (ns, not significant). **(C)** Conidiospore formation was observed under a light microscope. Scale bar, 50 μm. **(D)** A statistical analysis of conidiation for the WT, the Δ*ncl1* mutants, and the complemented strain is presented. Statistical significance was determined by one-way ANOVA followed by Tukey’s HSD test (**, $P < 0.01$; ns, not significant).

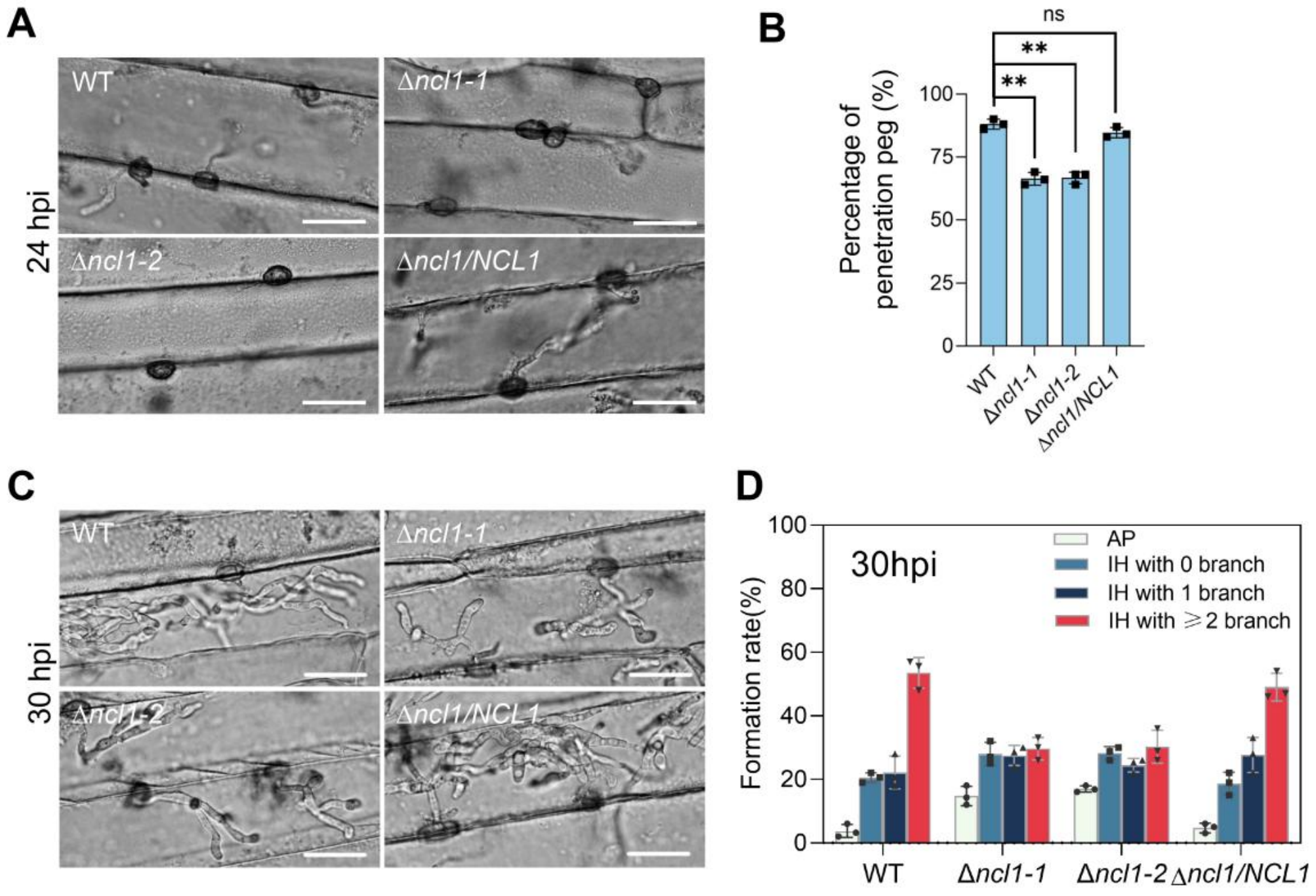

Figure S3. The expansion of invasive hyphae on the barley epidermis at 24 hpi and 30 hpi. (A) Penetration peg formation on the barley epidermis at 24 hpi for the WT, the Δ*ncl1* mutants, and the complemented strains. Scale bar, 20 μm. **(B)** Statistical analysis of appressorium penetration rates at 24 hpi. Data are means ± SD from 3 biological replicates (n > 100 appressoria per replicate). Statistical significance was determined by one-way ANOVA followed by Tukey's HSD test (**, $P < 0.01$; ns, not significant). **(C)** Infection hyphae formation on the barley epidermis at 30 hpi for the WT, the Δ*ncl1* mutant, and the complemented strain. Scale bar, 20 μm. **(D)** Statistical analysis of different types of infection hyphae (IH) at 30 hpi. Means and standard errors were calculated from more than 100 observations across three independent replicates.

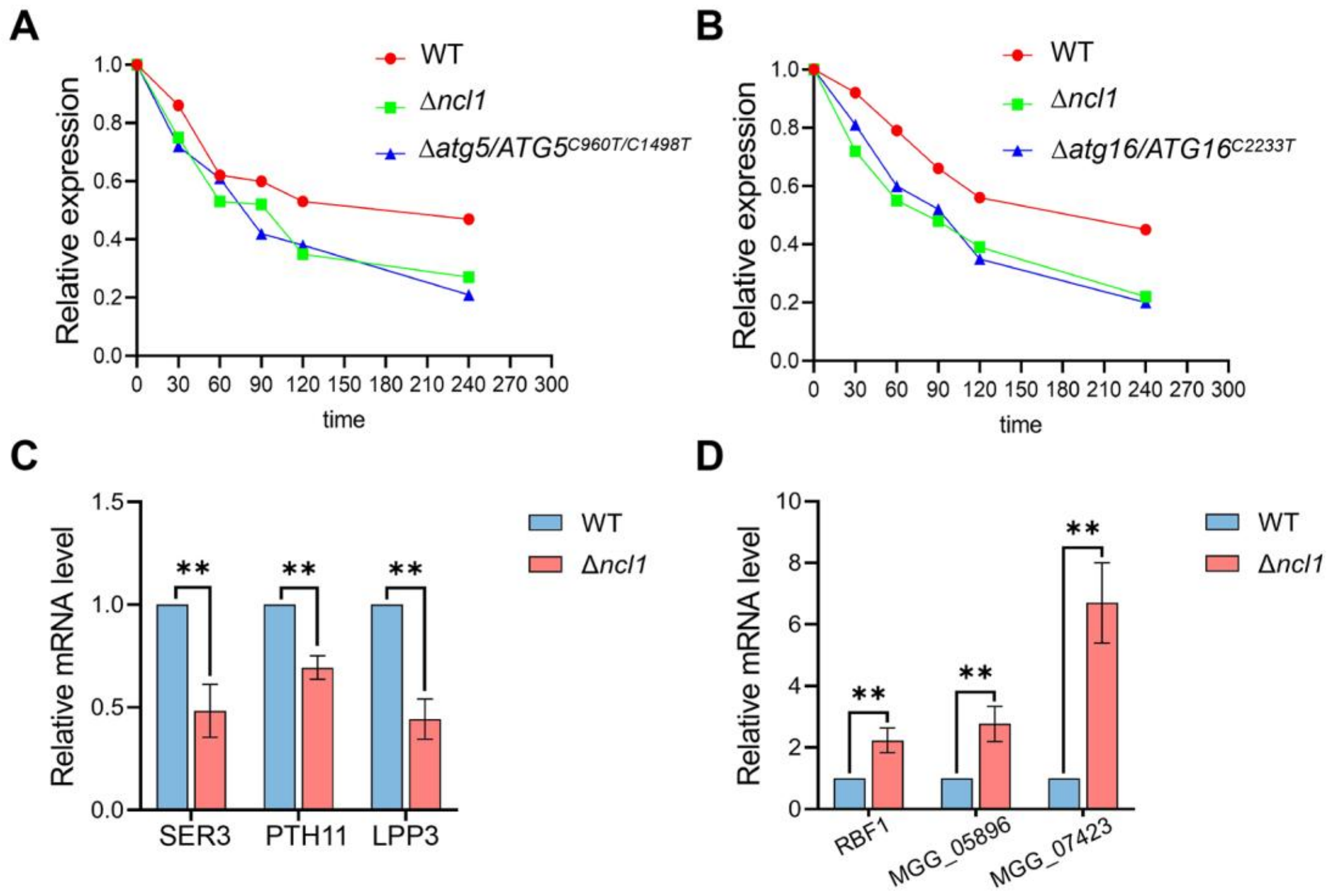

Figure S4. RNA decay assay and validation of RNA-seq data. (A, B) Vegetative hyphae of indicated strains were starved for nitrogen for 2 h and treated with 10 µg/mL actinomycin D to block transcription. Hyphae were harvested at 0, 30, 60, 90, 120, and 240 min post-treatment, and total RNA was extracted. *ATG5* and *ATG16* transcript levels were quantified by RT-qPCR. Data are means ± SEM from three independent replicates. **(C, D)** qRT-PCR analysis of six DEGs (three up-and three down-regulated). Data are means ± SE from 3 biological replicates (n = 3 technical replicates per biological replicate). Statistical significance was determined by two-sided unpaired Student’s t-test (**, $P < 0.01$).

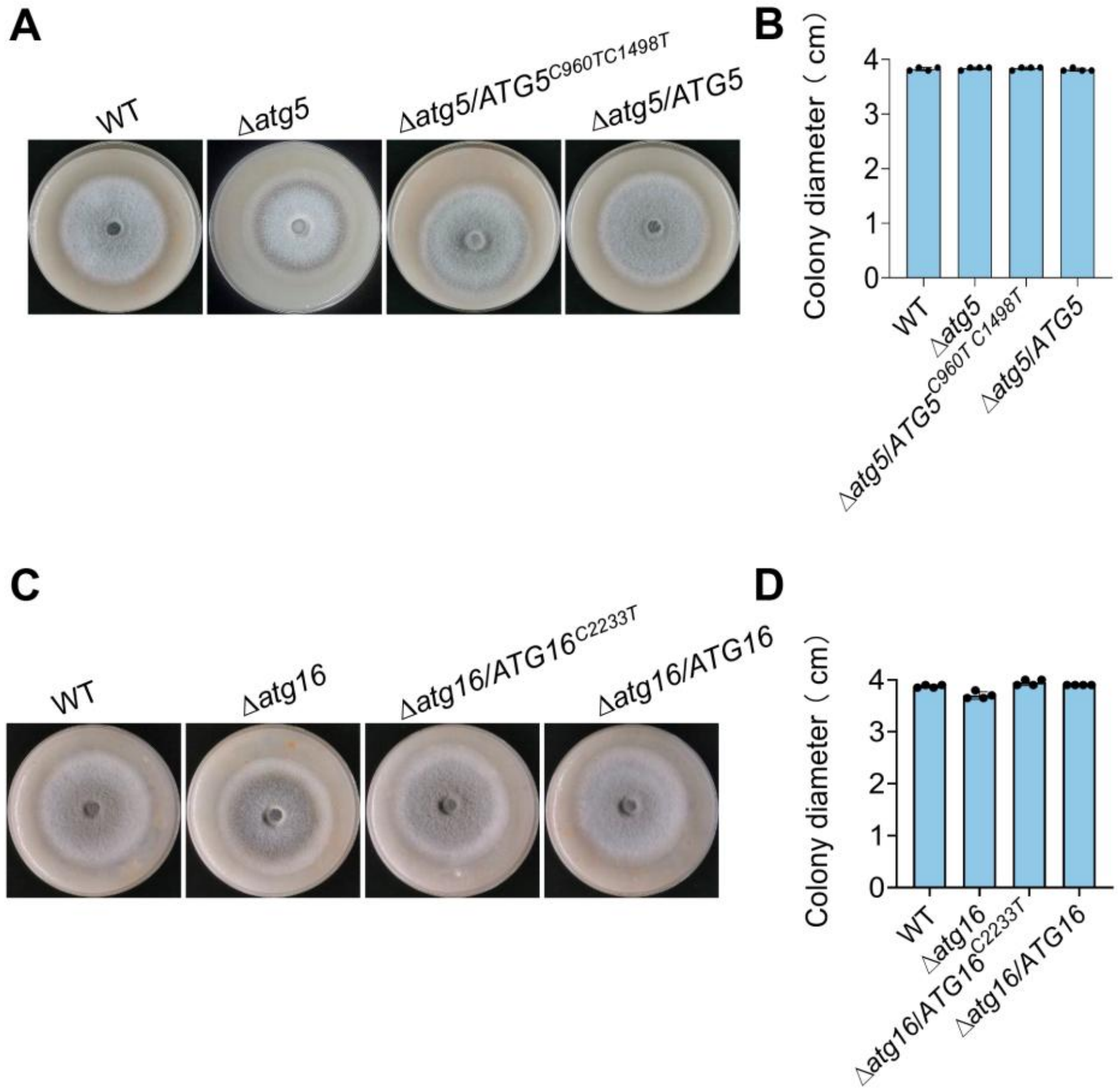

Figure S5. Colony growth analysis of autophagy-related mutants in *M. oryzae*. (A) Colony growth analysis of different *ATG5*-related mutant strains. The indicated strains were cultivated on oatmeal tomato agar (OTA) plates at 28°C for 5 days. **(B)** The colony diameters of different *ATG5*-related mutant strains were measured and statistically analyzed. Data are means ± SE from 3 biological replicates (n = 3 plates per replicate). Statistical significance was determined by one-way ANOVA followed by Tukey's HSD test (ns, not significant). **(C)** Colony growth analysis of different *ATG16*-related mutant strains. The indicated strains were cultivated on OTA plates at 28°C for 5 days. **(D)** The colony diameters of different *ATG16*-related mutant strains were measured and statistically analyzed. Data are means ± SE from 3 biological replicates (n = 3 plates per replicate). Statistical significance was determined by one-way ANOVA followed by Tukey's HSD test (ns, not significant).

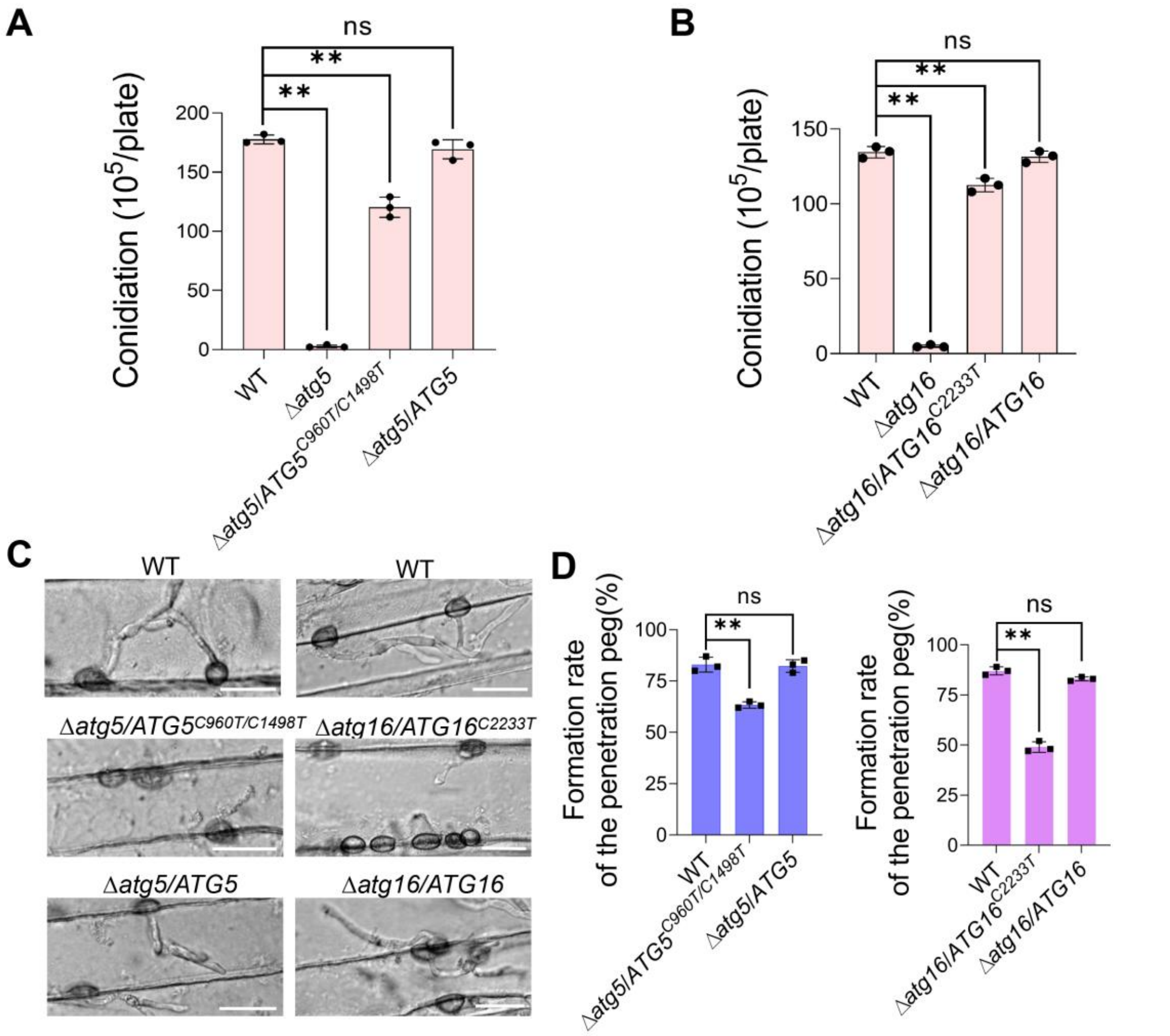

Figure S6. Conidiation in autophagy-related mutants of *M. oryzae*. (A) Statistical analysis of conidiation for *ATG5*-related mutant strains. Data are means ± SD from 3 biological replicates (n = 3 plates per replicate). Statistical significance was determined by one-way ANOVA followed by Tukey's HSD test (**, $P < 0.01$; ns, not significant). **(B)** Statistical analysis of conidiation for *ATG16*-related mutant strains. Data are means ± SD from 3 biological replicates (n = 3 plates per replicate). Statistical significance was determined by one-way ANOVA followed by Tukey's HSD test (**, $P < 0.01$; ns, not significant). **(C)** Penetration peg formation on the barley epidermis at 24 hpi of the different strains. Scale bar, 20 μm. **(D)** Statistical analysis of appressorium penetration rates at 24 hpi. Data are means ± SD from 3 biological replicates (n > 100 appressoria per replicate). Statistical significance was determined by one-way ANOVA followed by Tukey's HSD test (**, $P < 0.01$; ns, not significant).

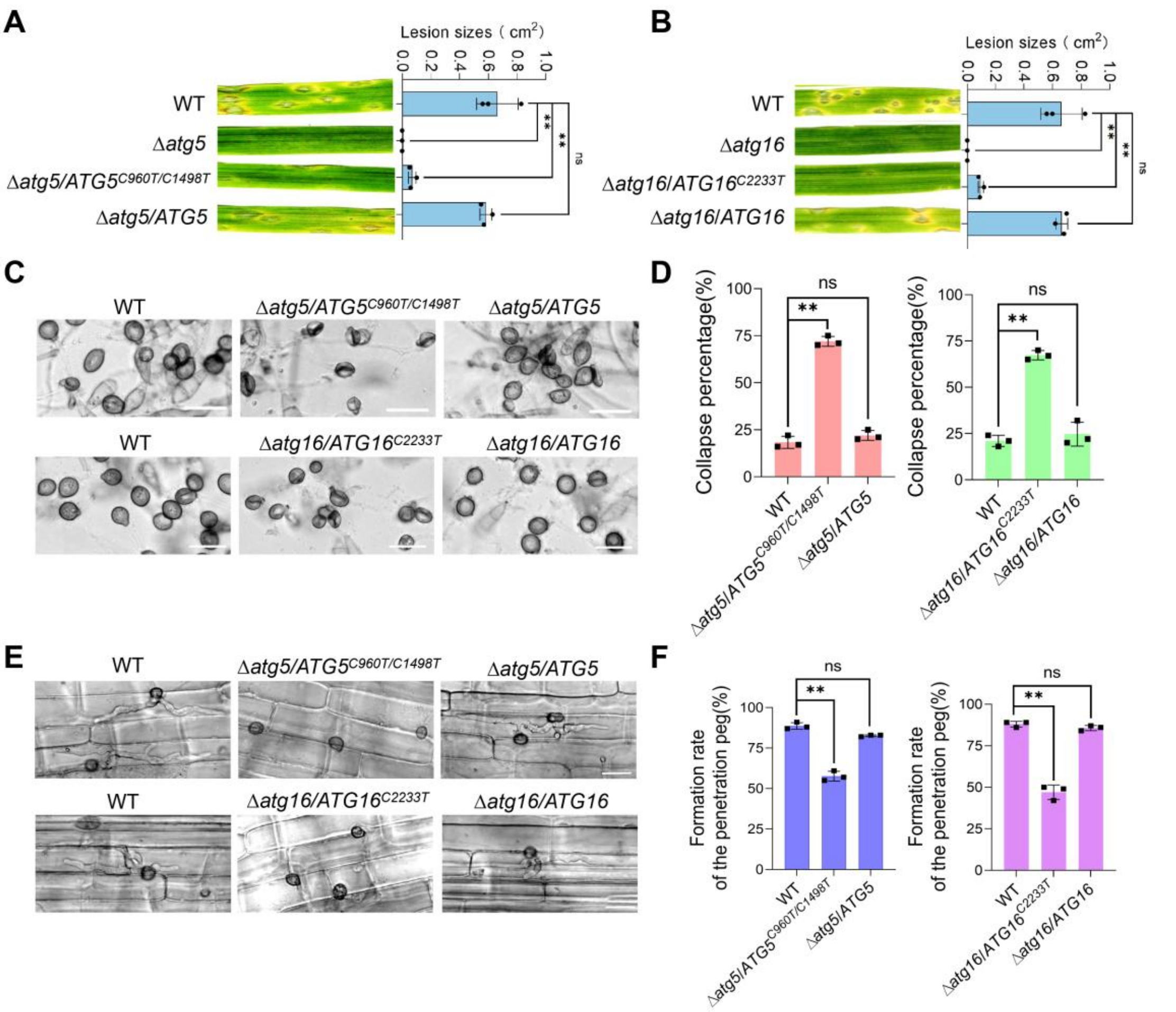

Figure S7. Impact of m^{5}C site mutations in *ATG5* and *ATG16* transcripts on appressorium development and pathogenicity. (A) Virulence test of the Δ*atg5/ATG5*$^{C960T/C1498T}$ strain on barley leaves. Data are means ± SD from 3 biological replicates (n = 3 leaves per replicate). Statistical significance was determined by one-way ANOVA followed by Tukey's HSD test (**, $P < 0.01$; ns, not significant). **(B)** Virulence test of the Δ*atg16/ATG16*^{C2233T} strain on barley leaves.Data are means ± SD from 3 biological replicates (n = 3 leaves per replicate). Statistical significance was determined by one-way ANOVA followed by Tukey's HSD test (**, $P < 0.01$; ns, not significant). **(C)** Morphological assessment of appressoria treated with 15% glycerol. Scale bar, 20 μm. **(D)** Percentage of appressorium collapse. Mature appressoria on hydrophobic surfaces were treated with 15% glycerol. Data are means ± SD from 3 biological replicates (n > 100 appressoria per replicate). Statistical significance was determined by one-way ANOVA followed by Tukey's HSD test (**, $P < 0.01$; ns, not significant). **(E)** Penetration peg formation on the rice leaf sheath at 24 hpi of the different strains. Scale bar, 20 μm. **(F)** Statistical analysis of appressorium penetration rates at 24 hpi. Data are means ± SD from 3 biological replicates (n > 100 appressoria

per replicate). Statistical significance was determined by one-way ANOVA followed by Tukey's HSD test (**, $P < 0.01$; ns, not significant).

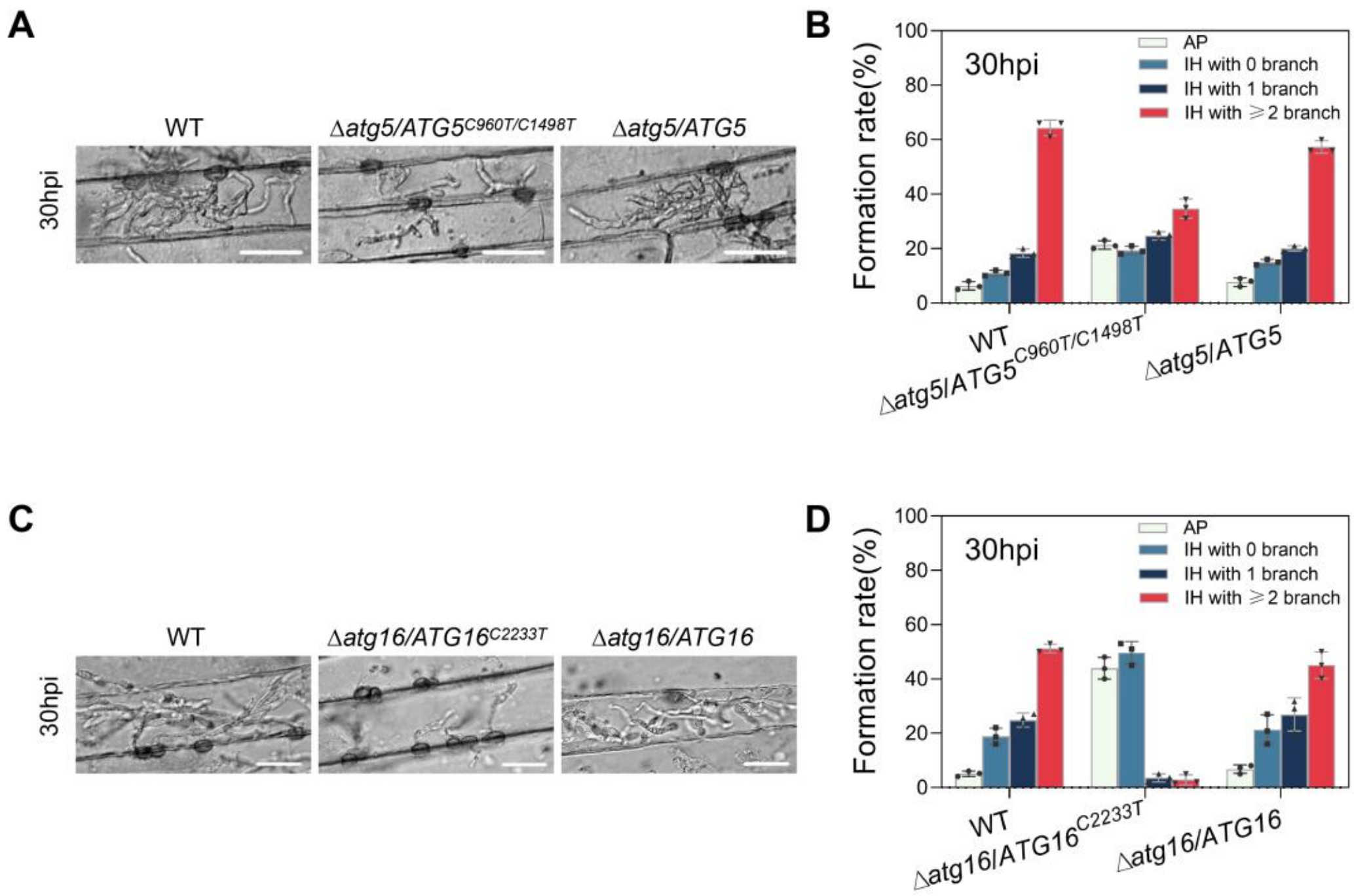

Figure S8. Invasive growth of autophagy-related mutants in *M. oryzae*. (A) Colonization of *ATG5*-related mutant strains on barley epidermis at 30 hpi. Scale bar, 20 µm. **(B)** Statistical analysis of the infection hyphae types for *ATG5*-related mutant strains at 30 hpi. Data are presented as means with standard errors from three independent experiments, with each experiment including more than 100 measurements (n > 100). **(C)** Colonization of *ATG16*-related mutant strains on barley epidermis at 30 hpi. Scale bar, 20 µm. **(D)** Statistical analysis of the infection hyphae types for *ATG16*-related mutant strains at 30 hpi. Data are presented as means with standard errors from three independent experiments, with each experiment including more than 100 measurements (n > 100).

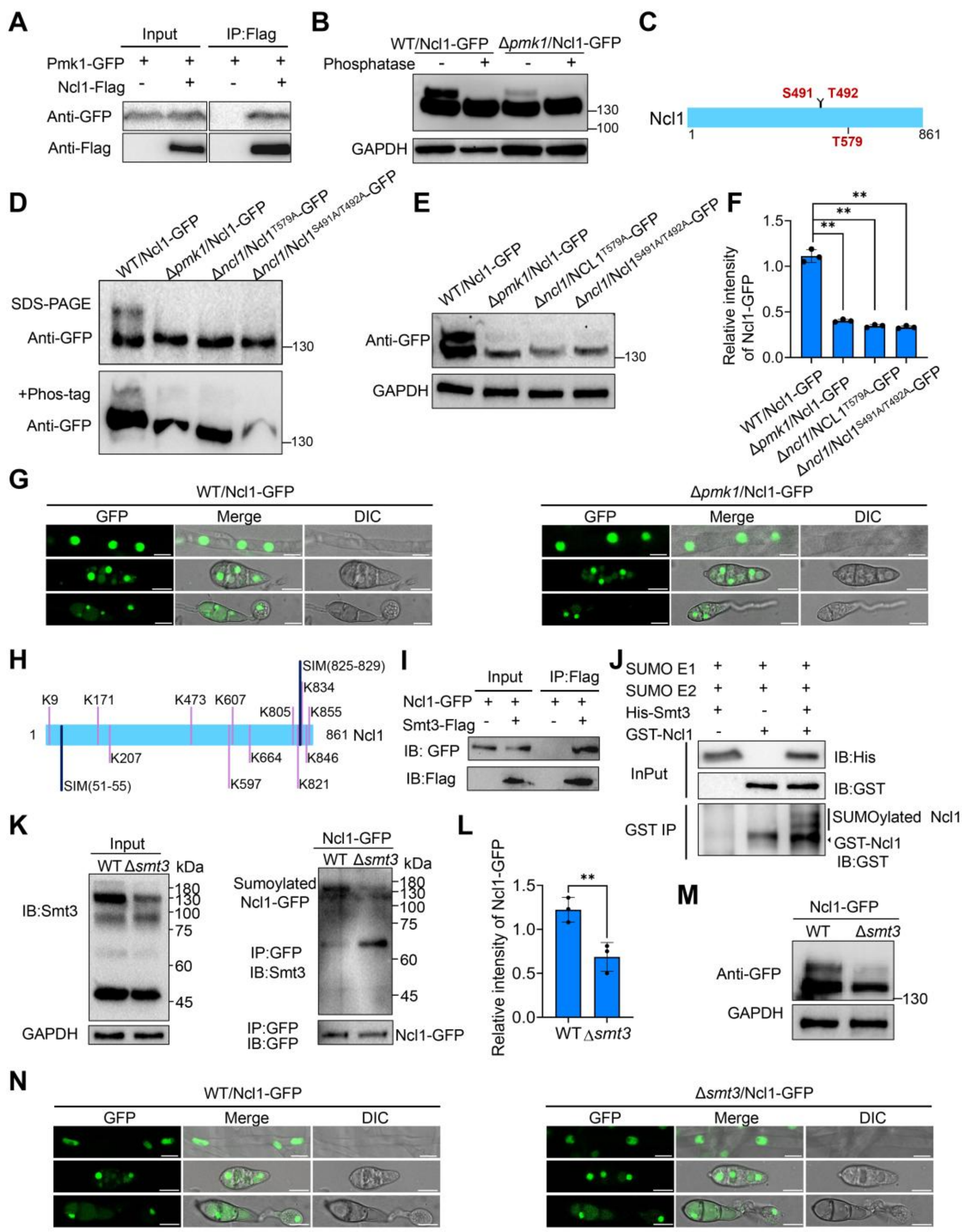

Figure S9. Regulation of Ncl1 function by Pmk1-mediated phosphorylation and Smt3-mediated sumoylation. (A) Co-immunoprecipitation assays verify the physical interaction between Ncl1 and Pmk1, using anti-Flag beads for immunoprecipitation and subsequent detection with anti-GFP and anti-Flag antibodies. **(B)** Determination of Ncl1-GFP protein levels in the WT and the Δ*pmk1* mutant after treatment with alkaline phosphatase at 37 °C for 1 hour. Anti-GAPDH was used as a loading control. **(C)** Phosphorylation sites on Ncl1 are identified using GC-MS. The workflow includes Ncl1 isolation, trypsin digestion, peptide derivatization, GC separation, MS

analysis for mass determination, and MS/MS for structural analysis to pinpoint phosphorylation sites, which are validated and quantified. **(D)** Phosphorylation levels of Ncl1 are assessed in different strains of *M. oryzae* mycelia. Proteins are separated using Mn^{2+}-Phos-tag SDS-PAGE to capture phosphorylated Ncl1 species and normal SDS-PAGE for total proteins, followed by immunoblotting with an anti-GFP antibody to detect Ncl1-GFP phosphorylation. **(E)** Determination of Ncl1-GFP protein levels in the Δ*pmk1* mutant and in strains with S491A/T492A and T579A mutations utilizing anti-GFP antibody. Anti-GAPDH was used as a loading control. **(F)** Relative intensity of Ncl1-GFP in WT and the Δ*pmk1* mutant by comparison with the internal control GAPDH. Statistical significance was determined by one-way ANOVA followed by Tukey's HSD test (**, $P < 0.01$). Error bars represent SE (**, $P < 0.01$). **(G)** Fluorescence microscopy analysis of Ncl1-GFP subcellular localization in wild-type and Δ*pmk1* mutant strains during mycelium, conidium, and appressorium development. Images captured at 10 μm scale. **(H)** *In silico* prediction of potential sumoylation sites and SUMO-interacting motifs within the Ncl1 protein sequence, as identified by GPS-SUMO 2.0 (https://sumo.biocuckoo.cn/). **(I)** Co-immunoprecipitation experiments demonstrating the interaction between Ncl1 and Smt3, utilizing anti-Flag beads for immunoprecipitation and detection with anti-GFP and anti-Flag antibodies. **(J)** *In vitro* sumoylation analysis of Ncl1. Components were incubated at 37 °C, and the reaction was purified and immunoblotted with anti-GST and anti-His antibodies. **(K)** *In vivo* sumoylation analysis of Ncl1-GFP in wild-type and Δ*smt3* backgrounds, utilizing anti-GFP beads for immunoprecipitation, sumoylation levels detected by anti-Smt3 antibody. Anti-GFP was used as a loading control. **(L)** Determination of Ncl1-GFP protein levels in WT and Δ*smt3* strains utilizing anti-GFP antibody. Anti-GAPDH was used as a loading control. Statistical significance was determined by two-sided unpaired Student's t-test (**, $P < 0.01$). **(M)** Relative intensity of Ncl1-GFP in WT and the Δ*smt3* mutant by comparison with the internal control GAPDH. Error bars represent SE (**, $P < 0.01$). **(N)** Fluorescence microscopy analysis of Ncl1-GFP subcellular localization in wild-type and Δ*smt3* mutant strains during mycelium, conidium, and appressorium development. Images captured at 10 μm scale.

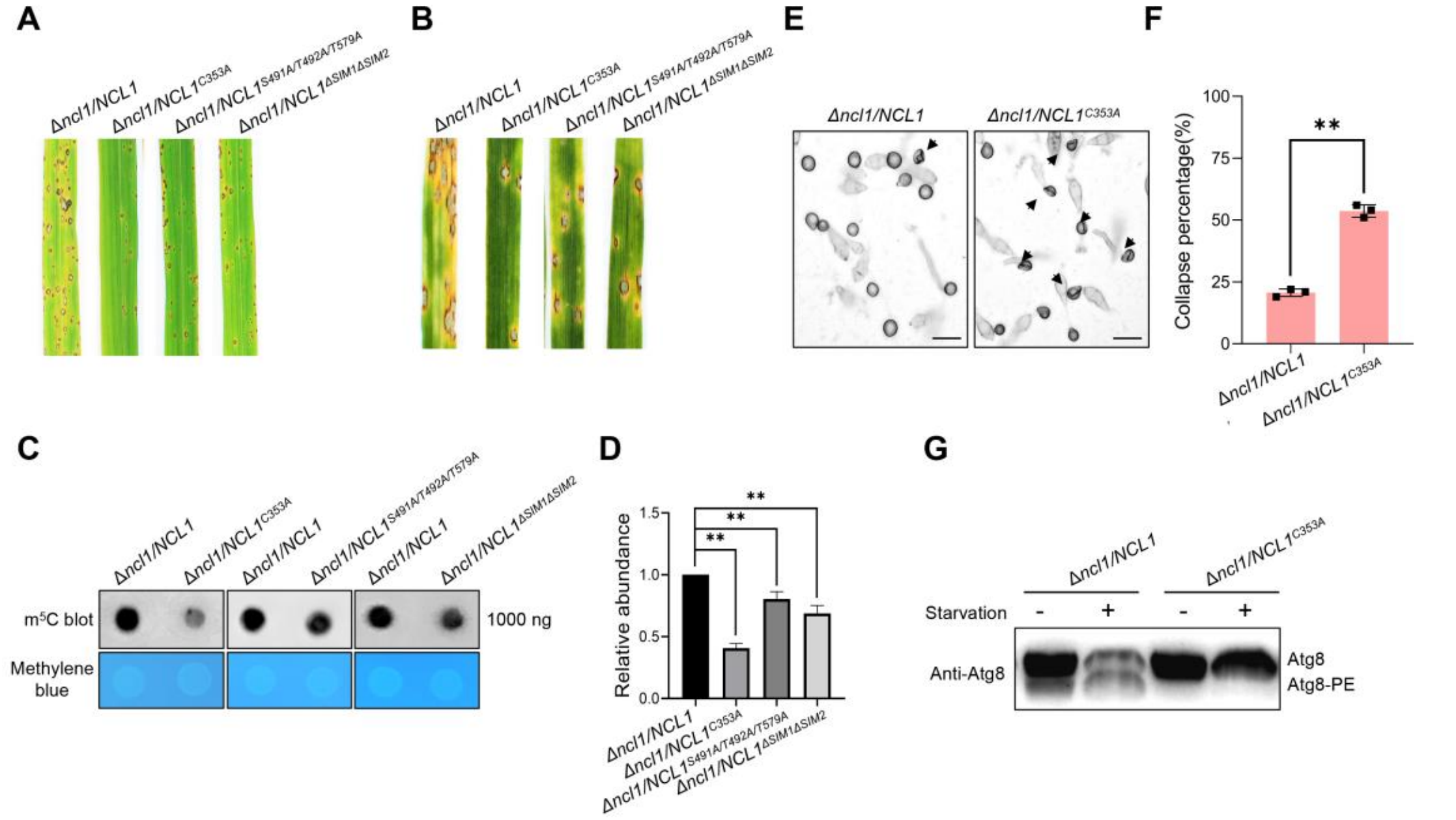

Figure S10. (A) Rice leaves were inoculated with conidial suspensions of different strains. Typical leaves were photographed at 5 dpi. **(B)** Barley seedlings were inoculated with conidial suspensions of different strains. Typical leaves were photographed at 5 dpi. **(C)** RNA dot blot analysis. m^5C levels in different strains were assessed using a specific m^5C antibody. Total RNA was extracted, spotted onto $Hybond^{TM}$-N^+ membranes, and hybridized with the m^5C antibody. Detection was performed using an enhanced chemiluminescence (ECL) system, with methylene blue staining serving as a loading control. **(D)** Quantification of RNA blot analysis. The intensity of the RNA blot signal, loaded at 1 μg, was quantified using ImageJ software. Data are means ± SD from 3 biological replicates. Statistical significance was determined by one-way ANOVA followed by Tukey's HSD test (**, $P < 0.01$). **(E)** Morphological changes in appressoria treated with 8% glycerol are shown. Arrows indicate collapsed appressoria. Scale bar, 20 μm. **(F)** The percentage of appressorium collapse after treatment with 8% glycerol is presented. Data are means ± SD from 3 biological replicates (**n > 100 appressoria per replicate). Statistical significance was determined by two-sided unpaired Student's t-test (**, $P < 0.01$). **(G)** Atg8-PE levels in different testing strains were detected in Urea-SDS-PAGE supplemented with 6M urea using anti-Atg8 antibody.

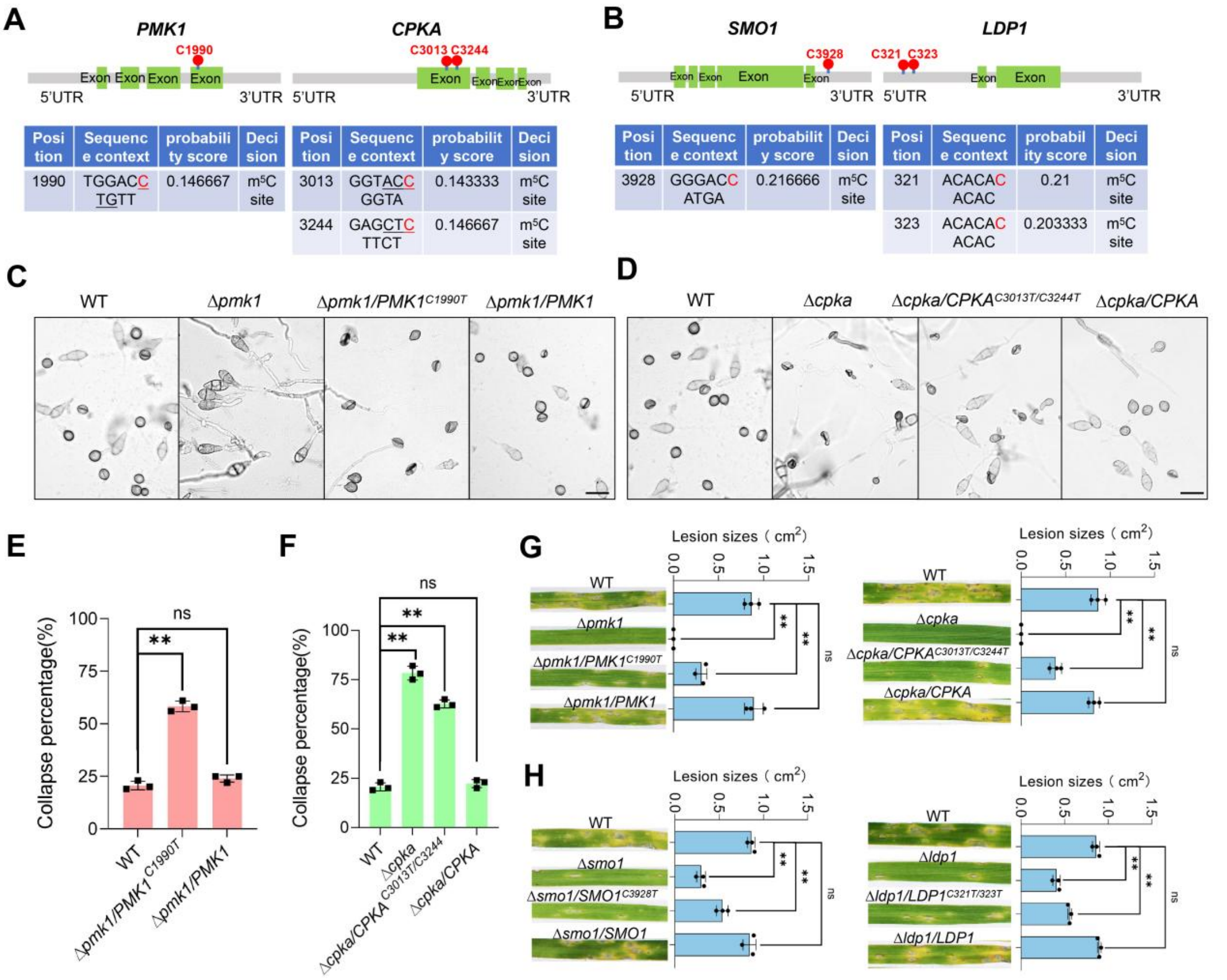

Figure S11. Functional validation of m^{5}C sites in non-*ATG* virulence-related genes. (A) Schematic of m^{5}C sites identified in *PMK1* (C1990) and *CPKA* (C3013/C3244) transcripts. Positions of C-to-T silent mutations are indicated. **(B)** Schematic of m^{5}C sites in *SMO1* (C3928) and *LDP1* (C321/C323) transcripts. **(C, D)** Morphological changes in appressoria treated with 15% glycerol are shown. Scale bar, 20 μm. **(E, F)** The percentage of appressorium collapse after treatment with 15% glycerol is presented. Data are means ± SD from 3 biological replicates (n > 100 appressoria per replicate). Statistical significance was determined by one-way ANOVA followed by Tukey's HSD test (**, $P < 0.01$; ns, not significant). **(G, H)** Virulence assays on barley leaves inoculated with conidial suspensions of the indicated strains. Lesion phenotypes were photographed at 5 dpi. Data are means ± SD from 3 biological replicates (n = 3 leaves per replicate). Statistical significance was determined by one-way ANOVA followed by Tukey's HSD test (**$P < 0.01$).

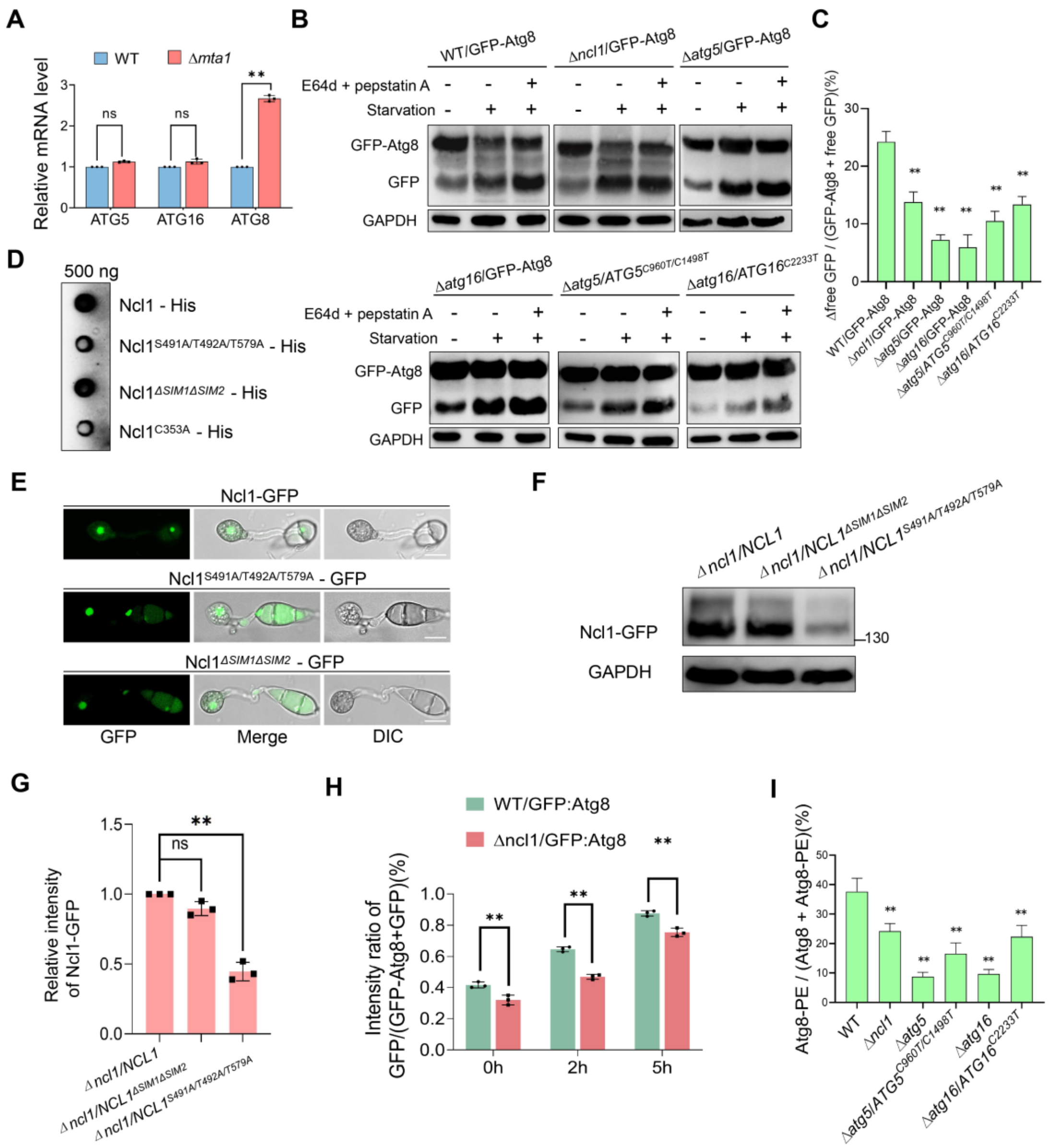

Figure S12. Supplementary validation of m^{6}A-m^{5}C crosstalk and Ncl1 catalytic activity. (A) qRT-PCR analysis of *ATG5*, *ATG16*, and *ATG8* transcript levels in WT and Δ*mta1* mutant during appressorium formation (6 hpi). Data are means ± SE from 3 biological replicates (n = 3 technical replicates per biological replicate). Statistical significance was determined by one-way ANOVA followed by Tukey's HSD test (**P < 0.01; ns, not significant). **(B)** Immunoblotting analysis of GFP-Atg8 processing in WT, Δ *ncl1*, Δ *atg5*, Δ *atg16*, Δ *atg5/ATG5*$^{C960T/C1498T}$, and Δ *atg16/ATG16*^{C2233T} strains treated with DMSO (mock) or 10 μg/mL E64d + 10 μg/mL pepstatin A for 3 h under nitrogen starvation. Anti-GAPDH served as a loading control. Full-length GFP-Atg8 and free GFP bands are indicated. **(C)** Quantitative analysis of autophagic flux. Flux was calculated as (free GFP in inhibitor-treated

group − free GFP in mock group)/total GFP in mock group. Data are means ± SE from 3 biological replicates. Statistical significance was determined by one-way ANOVA followed by Tukey's HSD test (**$P < 0.01$). **(D)** Detection of methylation of RNA m^5C by purified protein, in *vitro*. **(E)** Fluorescence microscopy of Ncl1-GFP, Ncl1$^{\Delta SIM1 \Delta SIM2}$-GFP, Ncl1$^{S491A/T492A/T579A}$-GFP localization localization during appressorium development. Scale bar, 10 μm. **(F)** Western blot analysis of Ncl1-GFP protein levels in Δ*ncl1*/NCL1, Δ*ncl1*/*NCL1*$^{\Delta SIM1 \Delta SIM2}$, and Δ*ncl1*/NCL1$^{S491A/T492A/T579A}$ strains. Anti-GAPDH served as a loading control. **(G)** Relative intensity of of Ncl1-GFP, Ncl1$^{\Delta SIM1 \Delta SIM2}$-GFP, Ncl1$^{S491A/T492A/T579A}$-GFP. Statistical significance was determined by one-way ANOVA followed by Tukey's HSD test (**, $P < 0.01$; ns, not significant). (H) Intensity ratio of GFP/(GFP-Atg8+GFP) in different testing strains as described in Figure 5M. Data are means ± SD from 3 biological replicates. Statistical significance was determined by two-sided unpaired Student's t-test (**, $P < 0.01$). (I) Quantitative analysis of Atg8-PE levels in Figure 6J. Data are means ± SE from 3 biological replicates. Statistical significance was determined by one-way ANOVA followed by Tukey's HSD test (**$P < 0.01$).

Supplemental Table 1 Significant reduced methylated peaks in mRNA of the Δ*ncl1* mutant.

Supplemental Table 2 Differentially expressed genes in the Δ*ncl1* mutant compared to the wild-type.

Supplemental Table 3 m^5C-seq results for transcripts of the autophagy-related genes.

Supplemental Table 4 Fungal strains used in this study.

Supplemental Table 5 Plasmids used in this study.
